# HTLV-1 Tax Functions as a Ubiquitin E3 Ligase for Direct IKK Activation via Synthesis of Mixed-Linkage Polyubiquitin Chains

**DOI:** 10.1371/journal.ppat.1005584

**Published:** 2016-04-15

**Authors:** Chong Wang, Wenying Long, Chao Peng, Lin Hu, Qiong Zhang, Ailing Wu, Xiaoqing Zhang, Xiaotao Duan, Catherine C. L. Wong, Yuetsu Tanaka, Zongping Xia

**Affiliations:** 1 Life Sciences Institute and Innovation Center for Cell Signaling Network, Zhejiang University, Hangzhou, Zhejiang, China; 2 National Center for Protein Science Shanghai, Institute of Biochemistry and Cell Biology, Shanghai Institutes for Biological Sciences, Chinese Academy of Sciences, Shanghai, China; 3 Shanghai Science Research Center, Chinese Academy of Sciences, Shanghai, China; 4 State Key Laboratory of Toxicology and Medical Countermeasures, Beijing Institute of Pharmacology and Toxicology, Beijing, China; 5 Department of Immunology, Graduate School of Medicine, University of the Ryukyus, Nishihara-cho, Okinawa, Japan; Imperial College London, UNITED KINGDOM

## Abstract

The HTLV-1 oncoprotein Tax plays a key role in CD4^+^ T cell transformation by promoting cell proliferation and survival, mainly through permanent activation of the NK-κB pathway and induction of many NF-κB target genes. Elucidating the underlying molecular mechanism is therefore critical in understanding HTLV-1-mediated transformation. Current studies have suggested multiple but controversial mechanisms regarding Tax-induced IKK activation mainly due to blending of primary Tax-induced IKK activation events and secondary IKK activation events induced by cytokines secreted by the primary Tax-induced IKK-NF-κB activation events. We reconstituted Tax-stimulated IKK activation in a cell-free system to dissect the essential cellular components for primary IKK activation by Tax and studied the underlying biochemical mechanism. We found that Tax is a putative E3 ubiquitin ligase, which, together with UbcH2, UhcH5c, or UbcH7, catalyzes the assembly of free mixed-linkage polyubiquitin chains. These free mixed-linkage polyubiquitin chains are then responsible for direct IKK activation by binding to the NEMO subunit of IKK. Our studies revealed the biochemical function of Tax in the process of IKK activation, which utilizes the minimal cellular ubiquitination components for NF-κB activation.

## Author Summary

Human T-cell leukemia virus type 1 (HTLV-1) is the etiologic agent of tropical spastic paraparesis/HTLV-1-associated myelopathy (TSP/HAM), a distinct neurological disorder with inflammatory symptoms and incomplete paralysis of the limbs, and adult T-cell leukemia/lymphoma (ATL), a highly aggressive malignant proliferation of CD4^+^ T lymphocytes. Both TSP/HAM and ATL are mainly driven by the activation of IκB kinase (IKK)-NF-κB stimulated by HTLV-1 oncoprotein Tax. The molecular mechanism by which Tax activates IKK remains unclear. Here, we found that Tax is an E3 ubiquitin ligase, which, together with its cognate ubiquitin-conjugating enzymes (E2s) UbcH2, UhcH5c, or UbcH7, catalyzes the assembly of unanchored free mixed-linkage polyubiquitin chains. The polyubiquitin chains can activate IKK complex directly by binding to the NEMO subunit. Our studies uncovered the essential cellular factors hijacked by HTLV-1 for infection and pathogenesis, as well as the biochemical function and the underlying mechanism of Tax in the process of IKK activation. Our work might shed light on potential development of therapeutics for TSP/HAM and ATL.

## Introduction

Human T-cell leukemia virus type 1 (HTLV-1), the first human oncogenic retrovirus that was originally described in 1980 [1], has been received much scientific attention due to its ability to transform primary T-lymphocytes in cell culture and its association with adult T-cell leukemia/lymphoma (ATL), a highly aggressive malignant proliferation of CD4^+^ T lymphocytes, and with tropical spastic paraparesis/HTLV-1-associated myelopathy (TSP/HAM), a distinct neurological disorder with inflammatory symptoms and incomplete paralysis of the limbs [2, 3].

HTLV-1 possesses an open reading frame (ORF) encoding a transactivator Tax that is critical to the viral life cycle for proviral transcription from the viral long terminal repeat (LTR) promoter [4]. Numerous studies have shown that it is also this Tax protein that is essential for mediating malignant T cell transformation by HTLV-1. Tax can transform rodent fibroblasts and human primary T cells in the presence of IL-2 [5]. In addition, Tax-transformed lymphoid cells and fibroblasts form tumors when inoculated into immunodeficient nude mice [6, 7], and Tax-based transgene expression induces an ATL-like syndrome in the T cell compartment in mice [8] and plasmatocyte proliferation in *Drosophila* [9]. Moreover, HTLV-1 genome without Tax loses its transformation ability [10]. Tax mutant that is defective in NF-κB activation loses the ability to transform T cells [11] and shows defect in cutaneous disease development in transgenic mice [12]. Additionally, increased studies have shown the minus strand of HTLV-1 encodes a bZIP protein HBZ that is critical for promoting proliferation of ATL cells [13].

Tax exerts a variety of activities in cells and undergoes heavy post-translational modifications such as phosphorylation, ubiquitination, sumoylation, and acetylation to control or modulate its cellular activities [14]. Tax interacts with more than 100 host cell proteins [15] and engages multiple signaling pathways such as activation of cAMP response element-binding protein (CREB), NF-κB, serum response factor (SRF) and inactivation of the tumor suppressor gene p53 [16]. Activation of such cellular proliferation-promoting pathways in turn induces a diverse array of genes encoding proliferative cytokines, cytokine receptors, co-stimulatory molecules as well as survival proteins [17]. Among these pathways, activation of NF-κB is arguably the most critical for Tax-associated cellular transformation and human diseases [18].

The NF-κB transcription factors include five members: RelA/p65, c-Rel, RelB, p105(p50), and p100(p52). These five members can form dimers with one another and bind to target DNA sequences called κB sites to modulate gene expression. In most un-stimulated cells, the NF-κB complexes are retained in the cytoplasm and inactive due to their binding by inhibitory IκB proteins (IκBα, IκBβ, IκBε, etc.) [19]. Upon activation, IκB proteins are phosphorylated, ubiquitinated and then degraded by the proteasome leading to release and translocation of NF-κB into the nucleus. Phosphorylation of IκBs is mediated by the IKK kinase complex, which consists of two active kinase subunits, IKKα and IKKβ, and the regulatory scaffolding subunit IKKγ (also called NEMO) [20]. In the TNFR and IL-1R/TLR activated NF-κB pathways, IKK activation requires an upstream kinase TGF-β-activating kinase 1 (TAK1) and adaptor proteins TRAFs such as TRAF6 [21]. In the case of TRAF6, it functions as an E3 ubiquitin ligase, together with Ubc13/Uev1, to catalyze assembly of K63-linked polyubiquitin (polyUb) chains to mediate TAK1 activation [22].

Activation of NF-κB by Tax also depends on IKK. Tax was shown to interact directly with IKKγ and induce its oligomerization [23–26]. Overexpression of Tax fusion protein to either IKKα or IKKβ was shown to be sufficient for IKK activation [27]. Tax was also found to localize to the lipid rafts, to where it recruited IKK for its persistent activation, which mechanism was further strengthened by cell adhesion molecule 1 (CADM1) [28, 29]. These studies suggest Tax and IKKγ interaction is important for Tax-mediated IKK activation [17]. Tax was also shown to interact with TAK1, the upstream kinase of IKK in the IL-1R/TLR signaling pathways, and this interaction was shown to mediate TAK1 interaction with IKK facilitating its activation [30]. In addition, MEKK1, NIK and Tpl2 were reported to be the putative IKK kinases for Tax-induced IKK activation [31–34]. However, by using various knockout MEF and siRNA-based knockdown cells, other studies illustrated those kinases were not required for Tax-induced IKK activation [35, 36]. Nonetheless, Tax alone was not sufficient for direct IKK activation and required other cellular factors [37].

It is generally recognized that Tax activation of IKK-NF-κB requires ubiquitination events, although there are debates as to what the ubiquitin-conjugating enzyme (E2s) and ubiquitin ligase (E3) are, and what the ubiquitination targets and the underlying mechanisms are. It was initially demonstrated that the E2 enzyme Ubc13, together with TRAF2, 5, or 6, promotes lysine-63 (K63) polyubiquitination of Tax, which targets IKK to centrosome thus promoting IKK activation [38, 39]. Tax also induces K63 polyubiquitination of NEMO for IKK activation [40]. A more recent study suggested that Tax stimulates E3 ligase RNF8, together with Ubc13/Uev1 and 2, to assemble K63 polyUb chains leading to activation of TAK1-IKK-NF-κB cascade [34]. In further support of this, the deubiquitinating enzyme (DUB) USP20 deubiquitinates Tax to negatively regulate activation of IKK [41]. But studies using RNAi-based knockdown and knockout MEF cells illustrated Ubc13 and TRAF6 are not required for Tax-induced IKK activation [35, 37]. Although Tax induced K63-linked polyubiquitination of NEMO, this event was not required for IKK activation [37]. In agreement with this, the K63-specific DUB CYLD effectively removed polyUb chains from NEMO; but this didn’t affect Tax-induced IKK activation [35]. In addition to ubiquitination, sumoylation of Tax was also reported to be important for IKK-NF-κB activation [42, 43]. However, Tax mutant defective in sumoylation, was still able to activate IKK without difference from Tax wild-type (WT) [44].

Therefore, a general but convincing mechanism about Tax-induced IKK activation awaits further studies. Like other IKK-NF-κB activation events, Tax-dependent IKK-NF-κB activation induces a variety of cytokines such as TNFα and IL-1β [45]. These cytokines in turn activate IKK-NF-κB through an autocrine and paracrine fashion. So the primary IKK-NF-κB activation events triggered by Tax and the secondary IKK-NF-κB signaling events triggered by secreted cytokines are blended together. However, all the cell- and animal-based studies cannot dissect the primary events from the secondary events. Therefore, these complications make it difficult to clearly define essential cellular factors and events for Tax-induced IKK activation.

To circumvent the complications brought about by the secondary IKK-NF-κB activation events, we developed a cell-free system to reconstitute IKK activation using recombinant Tax. This system allowed us to define critical cellular factors that are involved in primary events of Tax-induced IKK activation and to explore the underlying biochemical mechanism. We found that Tax is a putative E3 ubiquitin ligase, which, together with UbcH2, UhcH5c, or UbcH7, catalyzes the assembly of free unanchored mixed-linkage polyUb chains. These free mixed-linkage polyUb chains are then responsible for direct IKK activation by binding to the NEMO subunit of IKK. Our studies revealed the biochemical function and the underlying mechanism of Tax in the process of IKK activation, which utilizes the minimal cellular ubiquitination components for IKK-NF-κB activation.

## Results

### Cell-free reconstitution of IKK activation by Tax

**Fig 1:**
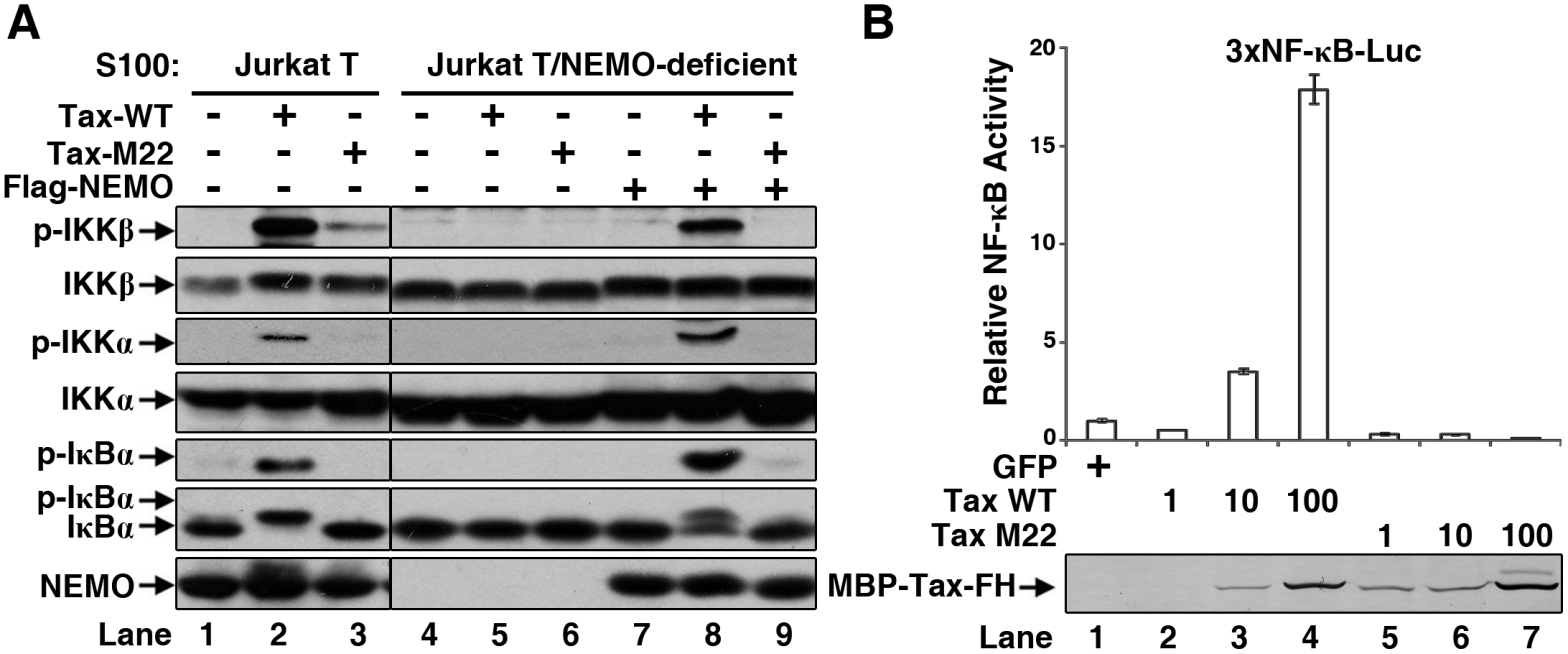
Tax activates NF-κB pathway. **(A)** Tax activates IKK in a cell-free system. Cell extracts (S100) of Jurkat T or NEMO-deficient Jurkat T cells were incubated with Tax WT or M22 (100 nM each), and ATP for 1 h at 30°C. Recombinant NEMO (100 nM) was added into the NEMO-deficient extracts to restore IKK activation. IKK activation was detected by phosphorylation of IKKα, IKKβ and its substrate IκBα. **(B)** Tax activates NF-κB in cells. 293T cells in 12-well-plates were transfected with 1, 10 or 100 ng of plasmids encoding Tax WT or M22, together with a NF-κB firefly-luciferase reporter and a renilla-luciferase reporter (internal control). The firefly-luciferase activity was measured 24 h later and normalized by renilla-luciferase. Tax protein expression was detected by immunoblotting with an anti-Tax antibody. FH: FLAG-10xHis tag.

To identify essential cellular components required for the primary IKK activation by Tax, we tried to reconstitute the activation process in a cell-free system. We generated recombinant Tax proteins by using baculoviral recombinant protein expression system (S1 Fig, left panel) and tested them in cellular cytosolic extracts S100 generated from Jurkat T cells. Incubation of Tax with S100 in the presence of ATP resulted in efficient IKK activation, as evidenced by the phosphorylation of IKKα, IKKβ and its physiological substrate IκBα (Fig 1A, lane 2). The mutant form of Tax, M22 (a double-site mutant originally reported to be defective in NF-κB activation) [46] [47], didn’t show detectable activity under the same conditions. The activation was dependent on the presence of NEMO, since there was no detectable IKK activity if we used S100 from NEMO-deficient Jurkat T cells (Fig 1A, lanes 4–6). But the activation was restored by adding recombinant NEMO back into the system (Fig 1A, lanes 7–9). Consistent with the IKK activation assay results *in vitro*, Tax WT but not the M22 stimulated the 3xκB-Luciferase (3xκB-Luc) reporter activity in a dose-dependent manner in 293T cells (Fig 1B). This set of experiments demonstrates that we have established a cell-free system to recapitulate Tax-dependent IKK activation in intact cells.

### Fractionation and identification of UbcH7 as an essential factor for Tax-induced IKK activation

**Fig 2:**
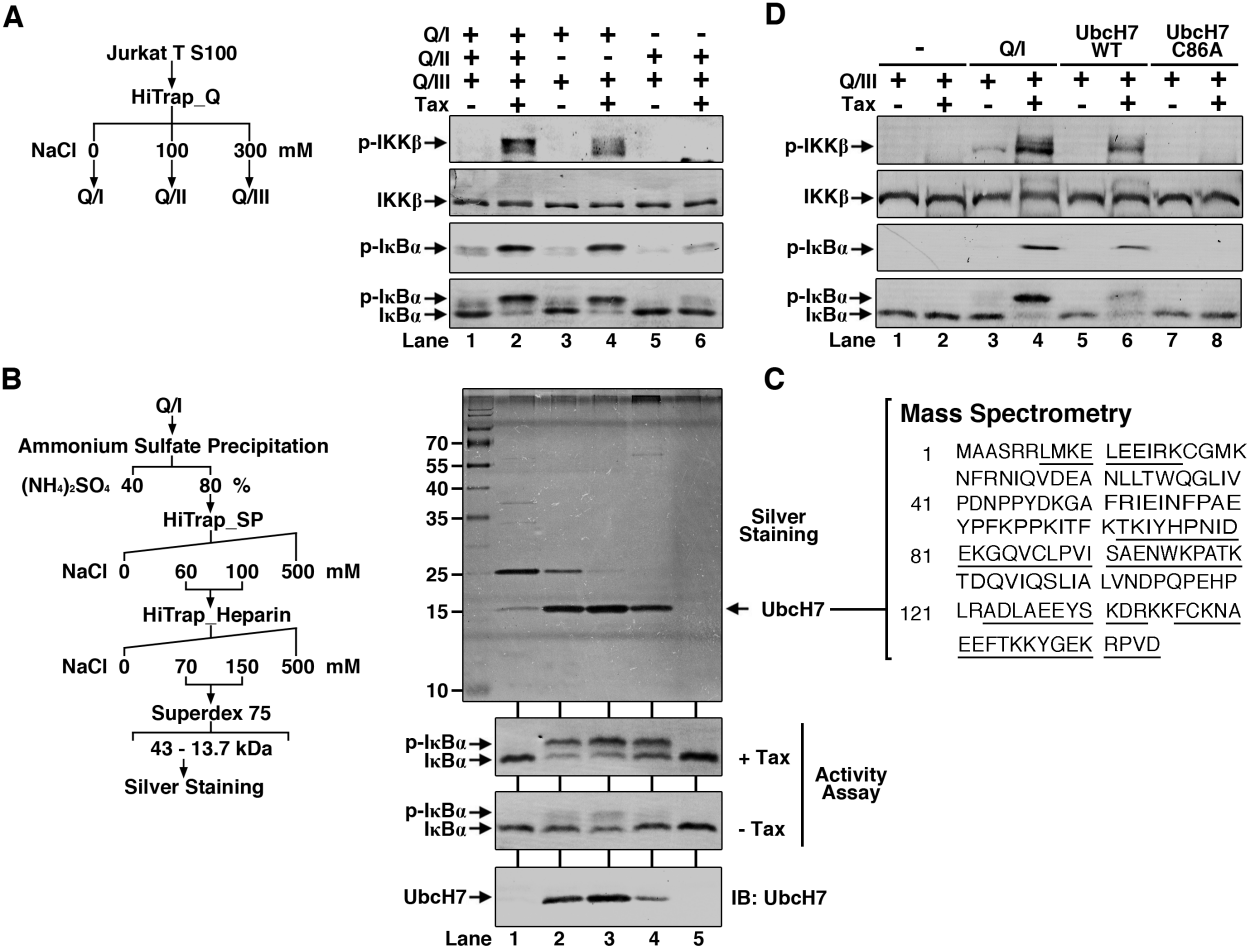
Purification and Identification of UbcH7. **(A)** Initial fractionation of S100. Jurkat T S100 was separated into 3 fractions on HiTrap Q column (left panel). The fractions were combined to perform the same *in vitro* IKK activation assay (right panel) as in Fig 1A. **(B)** Purification scheme of the activity in Q/I (left panel). Fractions from each step were incubated with Q/III to perform the *in vitro* IKK activation assay as in **(A)** to follow the activity. Proteins in the fractions of the last step were detected by silver staining. The band (about 15 kDa) corresponding to activity (Activity assay panel) in lane 3 on the silver staining gel was identified by mass spectrometry (MS) as shown in **(C)**. **(C)** Amino acid sequence of human UbcH7. Residues underlined are those detected by MS. **(D)** UbcH7 mediates IKK activation by Tax *in vitro*. The same *in vitro* assay as in **(A)** was performed except Q/I fraction was replaced with recombinant UbcH7 WT or the enzymatic-dead mutant (C86A).

We next fractionated the S100 into three fractions by using HiTRAP Q-sepharose column (GE Healthcare) through step elution with increasing concentration of NaCl (Fig 2A) and tested which fractions were required to restore IKK activation by Tax. Combination of Q/I and Q/III were sufficient for IKK activation by Tax (Fig 2A, lane 4; S2A Fig, lane 10). Q/I was also required for the activity, without which there was no IKK activation by Tax (Fig 2A, lane 6; S2A Fig, lane 12). These results suggest there is factor(s) in Q/I that supports Tax-dependent IKK activation.

We then focused on further fractionation for purification of the factor in Q/I. After five steps of conventional chromatograph (Fig 2B and S2B Fig), we achieved purification of the factor responsible for supporting Tax-dependent IKK activation. Fractions from the last Superdex-75 step were used for silver staining and IKK stimulatory activity testing (Fig 2B). By correlating the band intensities (Fig 2B, silver staining panel) and the IKK-stimulatory activity (Fig 2B, activity assay panel), the band present in fractions 2, 3 and 4 on the silver staining gel with an apparent molecular size of about 15 kDa is the most likely candidate. This band was excised and subjected to mass spectrometric analysis. The result suggested it is human UbcH7 (UBE2L3), a ub-conjugating enzyme E2 (Fig 2C).

To verify that UbcH7 is indeed the factor in Q/I that supports IKK activation by Tax, we expressed it in *E*.*coli* as a Hexahistidine (His_6_)-tagged recombinant protein and purified it to apparent homogeneity. We also expressed and purified its corresponding enzymatic activity dead mutant Cysteine-86 to Alanine (C86A) (S2C and S2D Fig). Similar to Q/I, recombinant UbcH7 activated IKK in the presence of Tax (Fig 2D). The C86A mutant was not able to stimulate Tax-dependent IKK activation, suggesting the enzymatic E2 activity is required to support Tax-dependent IKK activation (Fig 2D).

Together these data demonstrate that Tax-induced IKK activation is mediated by UbcH7 and depends on ubiquitination.

### Multiple E2s can confer Tax-dependent IKK activation

**Fig 3:**
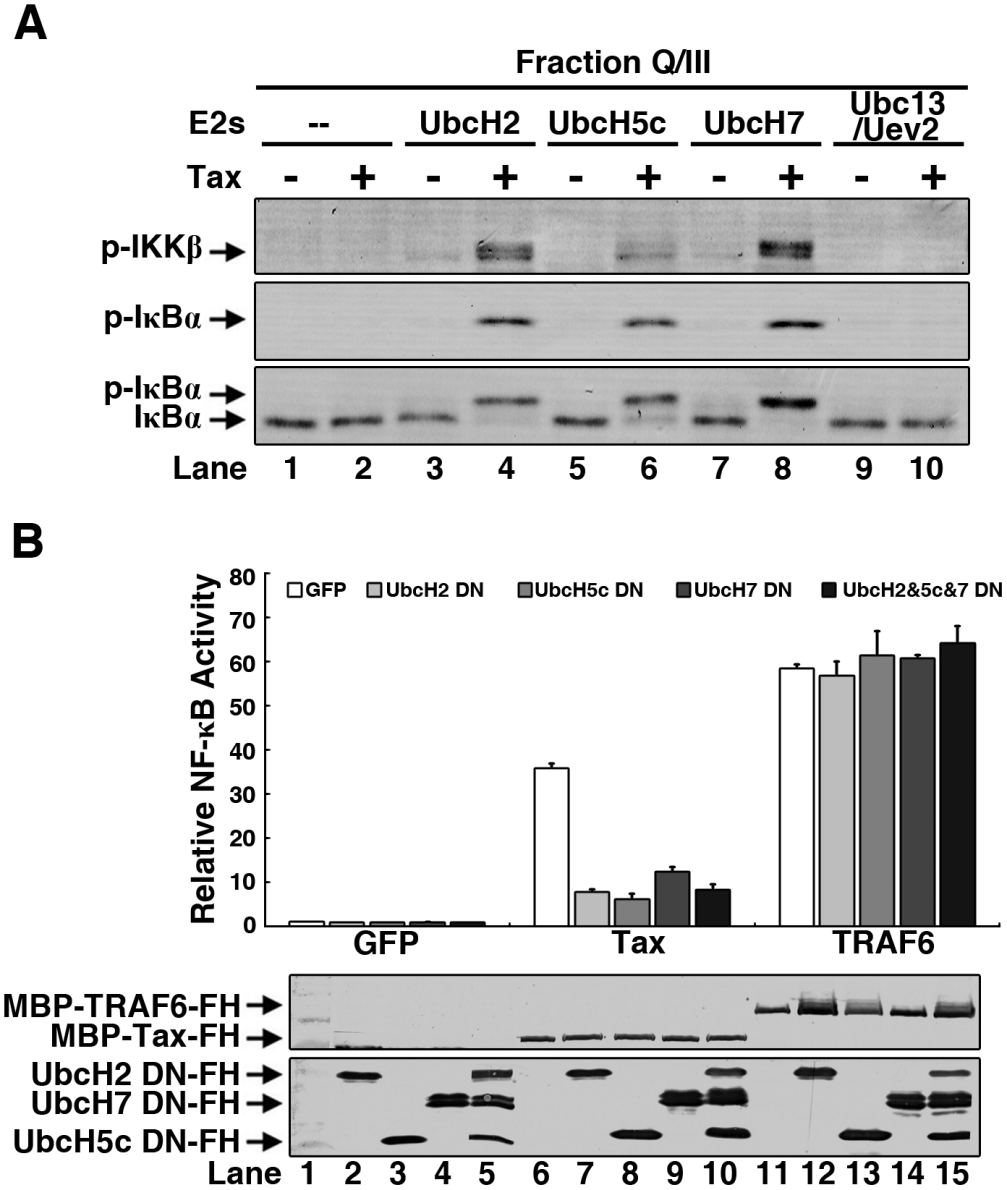
Multiple E2s confer Tax-dependent IKK activation. **(A)** Multiple E2s mediate IKK activation by Tax *in vitro*. The same *in vitro* assay as in Fig 2D was performed except UbcH7 was replaced with the indicated recombinant E2s. **(B)** E2 DN (dominant-negative) mutants impair IKK-NF-κB activation by Tax in cells. 293T cells were transfected with E2 DN mutants, or GFP as a control. After 6 hours, cells were transfected again with plasmids encoding Tax or TRAF6, together with a NF-κB firefly-luciferase reporter and a renilla-luciferase internal control. The luciferase activity was measured and normalized by renilla luciferase. Protein expression was detected by immunoblotting with an anti-FLAG antibody. FH: FLAG-10xHis tag.

Considering that many ubiquitination systems use several cognate E2s, we tested a panel of other E2s (S3A and S3B Fig) to check whether any of them could also support Tax-dependent IKK activation. As shown in Fig 3A and S3B Fig, in addition to UbcH7, two other E2s, UbcH2 (UBE2H) and UbcH5c (UBE2D3) were also capable of stimulating IKK activation by Tax. It is worth to note that the heterodimeric E2 complex, Ubc13/Uev2, specializing in the synthesis of K63 polyUb chains, was not able to activate IKK together with Tax (Fig 3A and S3B Fig). It has been well established that Ubc13/Uev1 (or Ubc13/Uev2) is a key E2 for IKK activation in the IL-1R and TLR pathways. There are also reports suggesting Ubc13 is involved in Tax-dependent IKK-NF-κB activation, although this requirement is currently under debate [35, 38, 39].

To determine whether the above-identified E2s are also required for IKK-NF-κB activation in living cells, we tested the effect on Tax-dependent NF-κB activation in the 3xκB-Luc reporter assay by transfecting their respective active site mutants into 293T cells. Transfection of Tax activated the 3xκB-Luc reporter. Remarkably, the activation was dramatically reduced by overexpression of these E2 Cysteine-to-Alanine (C-to-A) mutants, which presumably function as dominant-negative mutants (Fig 3B). In contrast, the three C-to-A mutants didn’t show any detectable effect on the reporter activity stimulated by TRAF6 overexpression (Fig 3B), which has been known to function together with Ubc13/Uev1 for IKK-NF-κB activation [48].

To further exclude the possibility that Ubc13 is able to support Tax-dependent IKK activation, we treated S100 with OspI or its active site mutant C62A and then tested it in the *in vitro* IKK activation assay. OspI has recently been shown to deamidate Gln100 of Ubc13 into Glu thus inactivating its E2 activity [49, 50]. As shown in S3C Fig, although both Tax and TRAF6 activated IKK as determined by phosphorylation of IKK and IκBα in the S100, only Tax but not TRAF6 still activated IKK in the S100 pre-treated with OspI. As a control, C62A mutant didn’t destroy the activity. Notably, only TRAF6 but not Tax treatment led to detectable phosphorylation and thus activation of TAK1 (S3C and S3E Fig). In agreement with the *in vitro* assay, in the 3xκB-Luc reporter assay in 293T cells, OspI only inhibited TRAF6 but not Tax-dependent reporter activity (S3D Fig).

### IKK activation by Tax is independent of TAK1 and TRAF6

Based on our previous experiences, Q/III fraction contains TAK1, TRAF6, IKK and its substrate IκBα. It has been reported that both TRAF6 and TAK1 are involved in Tax-dependent IKK activation [30]. To test this, we generated TAK1 and TRAF6 knockout (KO) cells by using CRISPR/Cas9-based genome editing technology in 293T cells and used them in the 3xκB-Luc reporter assays. KO of either TAK1 or TRAF6 didn’t have any effect on reporter activity by Tax overexpression, which displayed comparable activity to that in parental 293T cells (S3F Fig). As a positive control, KO of TAK1 abolished TRAF6-stimulated reporter activity. We also tested this directly by preparing S100 from 293T and TAK1 KO cells. Although S100 from 293T cells supported IKK activation by both Tax and TRAF6, S100 from TAK1 KO cells only supported IKK activation by Tax but not TRAF6 (S3E Fig). These results strongly demonstrate that activation of IKK by Tax doesn’t require TAK1 and TRAF6.

### Direct IKK activation by Tax in a ubiquitination-dependent manner

**Fig 4:**
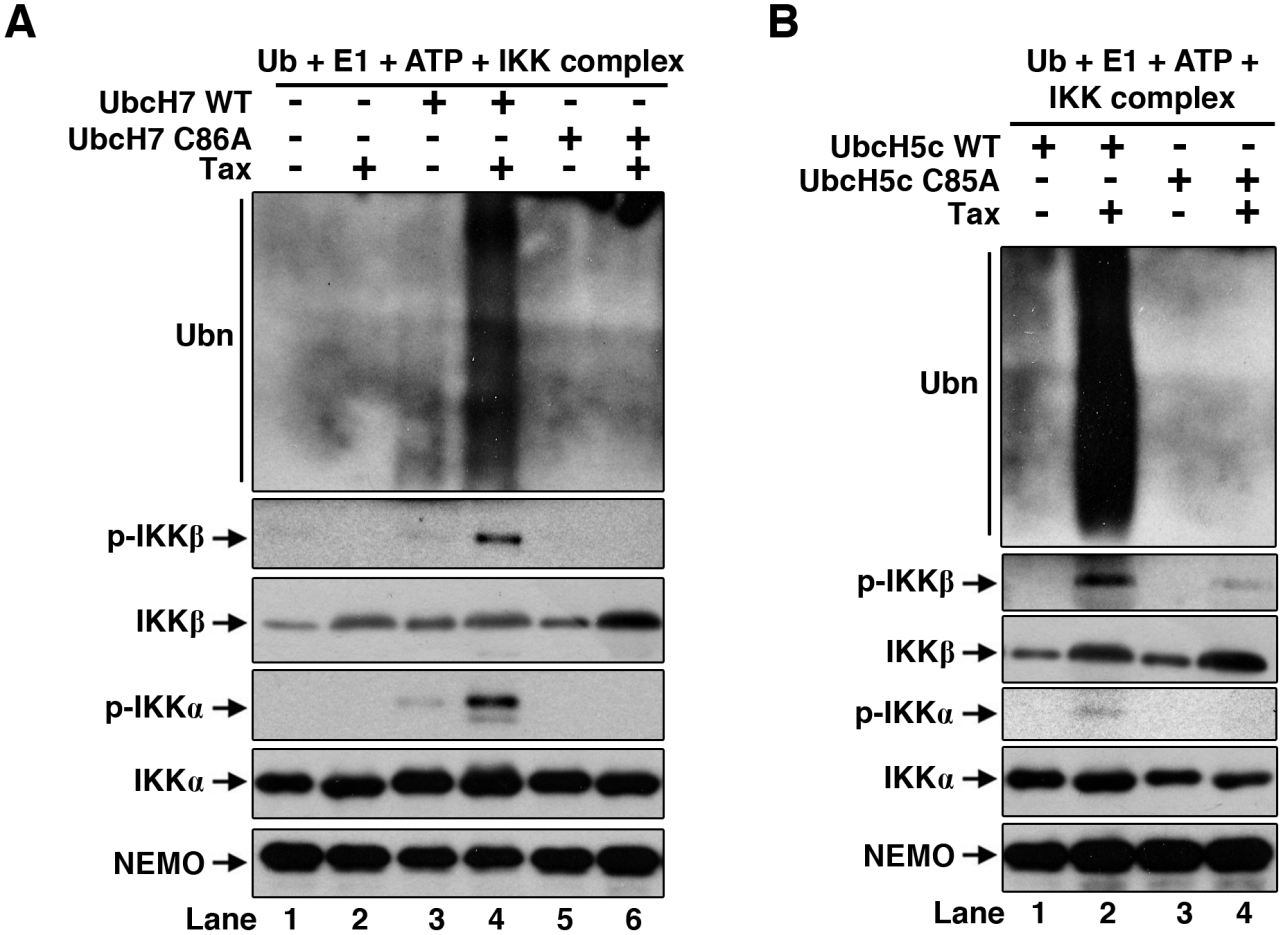
Direct IKK activation by Tax *in vitro*. **(A)** IKK complex purified from 293F/NEMO-FLAG knock-in cells was incubated with recombinant E1, Ub, UbcH7 WT or C86A mutant and Tax in ATP buffer at 37°C for 2 h. IKK activation was determined by phosphorylation of IKKα and IKKβ. Polyubiquitin chains were detected by anti-ubiquitin immunoblotting. IKKα, IKKβ and NEMO were also blotted. **(B)** The same assay as in **(A)** was performed except the UbcH7 was replaced by UbcH5c or its enzymatic-dead mutant C85A.

After exclusion of TAK1 and TRAF6 as the putative factors for Tax-dependent IKK activation in Q/III, we then tested if IKK complex itself is the only factor in Q/III for its activation by Tax. For this purpose, we generated a stable cell line expressing NEMO-FLAG by using CRISPR/Cas9-based knock-in in 293T cells, which was then used for IKK complex purification. Silver staining shows that the highly purified IKK complex contains IKKα, IKKβ and NEMO-FLAG (S4A Fig). We then tested if the IKK complex could be directly activated by Tax and UbcH7. As shown in Fig 4A, incubation of IKK, UbcH7 and Tax, together with E1, ubiquitin (Ub) and ATP, resulted in activation of IKK as determined by phosphorylation of both IKKα and IKKβ (Lane 4). Lack of either UbcH7 or Tax didn’t lead to significant IKK activation (Lanes 1–3). The activation depends on the E2 activity of UbcH7, since UbcH7 C86A didn’t support the activation. Similarly, replacement of UbcH7 by UbcH5c also activated IKK (Fig 4B). But the Ubc13/Uev2 was not able to facilitate IKK activation by Tax (S4B Fig). Interestingly, immunoblotting of the reaction products revealed presence of polyUb chains, which correlated very well with IKK activation in the case of UbcH7 and UbcH5c (Fig 4A and 4B, top panels). Combination of Tax and Ubc13/Uev2 also generated significant amount of polyUb chains, although there was no IKK activation (S4B Fig). Together, these data suggest that IKK itself is the only minimal factor in Q/III, which can be activated by Tax directly in a polyubiquitination-dependent manner.

### Tax is an E3 ubiquitin ligase

**Fig 5:**
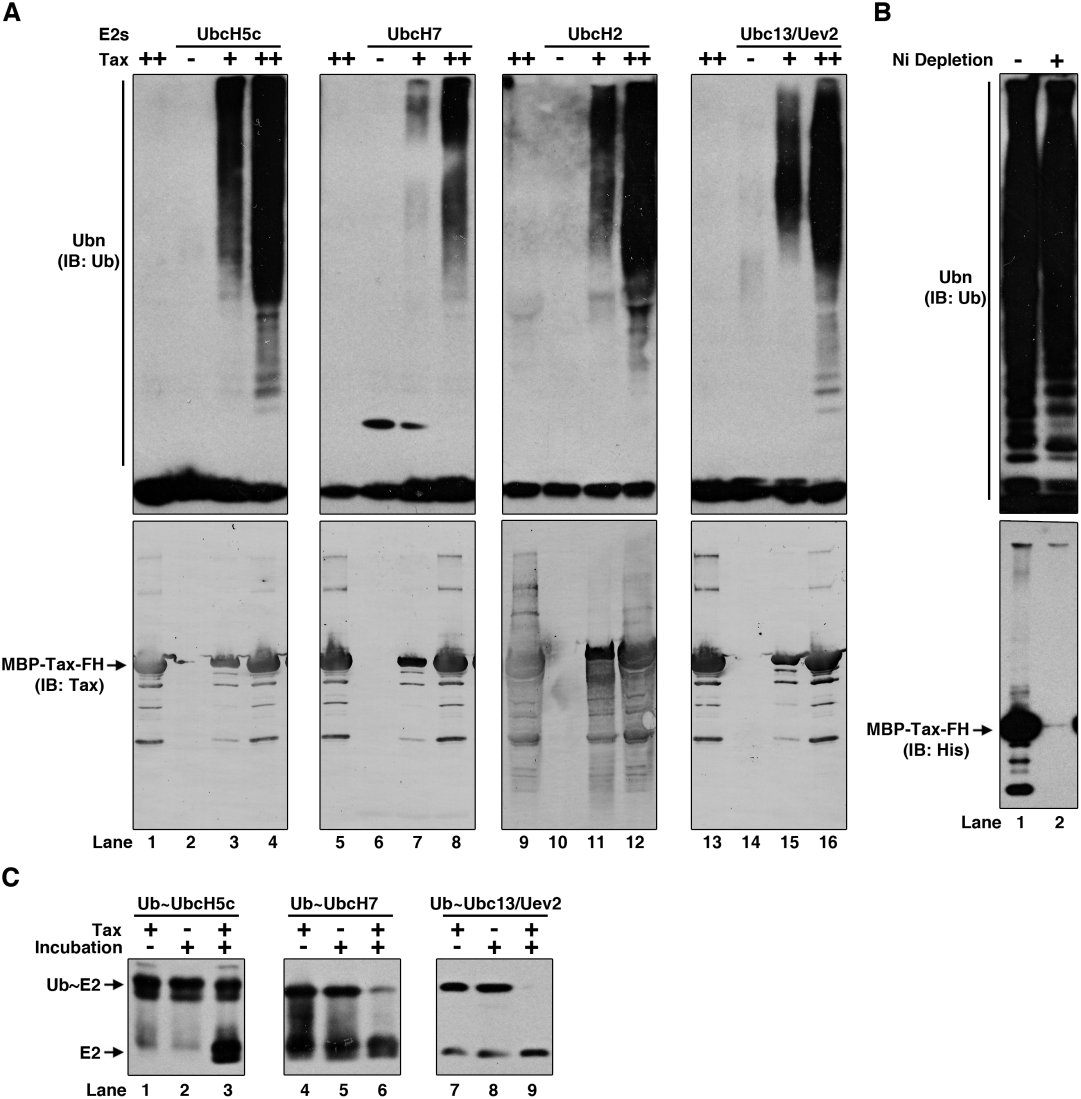
Tax shows E3 activity *in vitro*. **(A)** *In vitro* ubiquitination assay with Tax. Different concentrations of Tax protein were incubated with E1, Ub and indicated E2s in ATP buffer at 37°C for 2 h. The products were immunoblotted by anti-ubiquitin and anti-Tax antibodies. **(B)** The reaction mixtures in **(A)** by UbcH5c and Tax were incubated with Ni-NTA beads at 4°C for 2 h to immunodeplete His-tagged reaction components (E1, UbcH5c and Tax). The supernatant was immunoblotted by anti-ubiquitin and anti-Tax antibodies. **(C)** Ubiquitin discharging from Ub~E2s by Tax. Ub~E2 thioester intermediates (prepared as described in Materials and Methods) were incubated with Tax at 30°C for 15 min. Discharged E2s were detected by immunoblotting with their respective antibodies. FH: FLAG-10xHis tag.

The demonstration that highly purified IKK can be activated by Tax directly in a polyubiquitination-dependent manner prompted us to consider the possibility that Tax itself could be an E3 ubiquitin ligase. We tested this directly in two ways. Firstly, we carried out a typical ubiquitination assay *in vitro*. Incubation of E1, UbcH7, Ub and ATP didn’t lead to detectable polyUb signals. However, inclusion of Tax in the reaction led to significant amount of polyUb signals in a dose-dependent manner. Similar results were obtained when UbcH7 was replaced by UbcH5c, UbcH2 or Ubc13/Uev2. (Fig 5A, upper panels). It has been known that a few E2s such as UbcH5c can ubiquitinate a protein without the presence of any E3s. Immunoblotting of the reaction products using anti-Tax antibody didn’t show detectable ubiquitination of Tax (Fig 5A, bottom panels), demonstrating the polyUb signals were not due to polyUb conjugation of Tax protein itself and Tax didn’t simply function as a substrate but as an E3 ubiquitin ligase. These data also imply the polyUb chains are probably free unanchored polyUb chains. In further support of synthesis of free polyUb chains by Tax, we did affinity-depletion using Nickel beads against the ubiquitination reaction products to remove all the His-tagged proteins including E1, E2 and Tax that we added into the ubiquitination reaction. Immunoblotting of the supernatant after the depletion demonstrates that the His-tagged ubiquitination reaction components, especially Tax, have been removed to under detection level (Fig 5B, bottom panel). Under this condition, immunoblotting by using anti-Ub antibody showed comparable polyUb signals before and after the depletion, in agreement with our assumption that most, if not all, of the polyUb chains are free chains that are not conjugated to any proteins (Fig 5B, upper panel). Secondly, one feature of a typical E3 is its ability to promote turnover (discharge) of Ub from its cognate E2s when these E2s are loaded with Ub. So, we tested if Tax could do the same and found that Tax indeed promoted discharge of Ub from Ub~UbcH7, Ub~UbcH5c, and Ub~Ubc13 after incubation together (Fig 5C).

These two sets of experiments strongly suggest that Tax is a bona-fide E3 ubiquitin ligase, and some of its cognate E2s are UbcH7, UbcH5c, UbcH2 and Ubc13/Uev2.

### Tax catalyzes synthesis of free mixed-linkage polyUb chains

**Fig 6:**
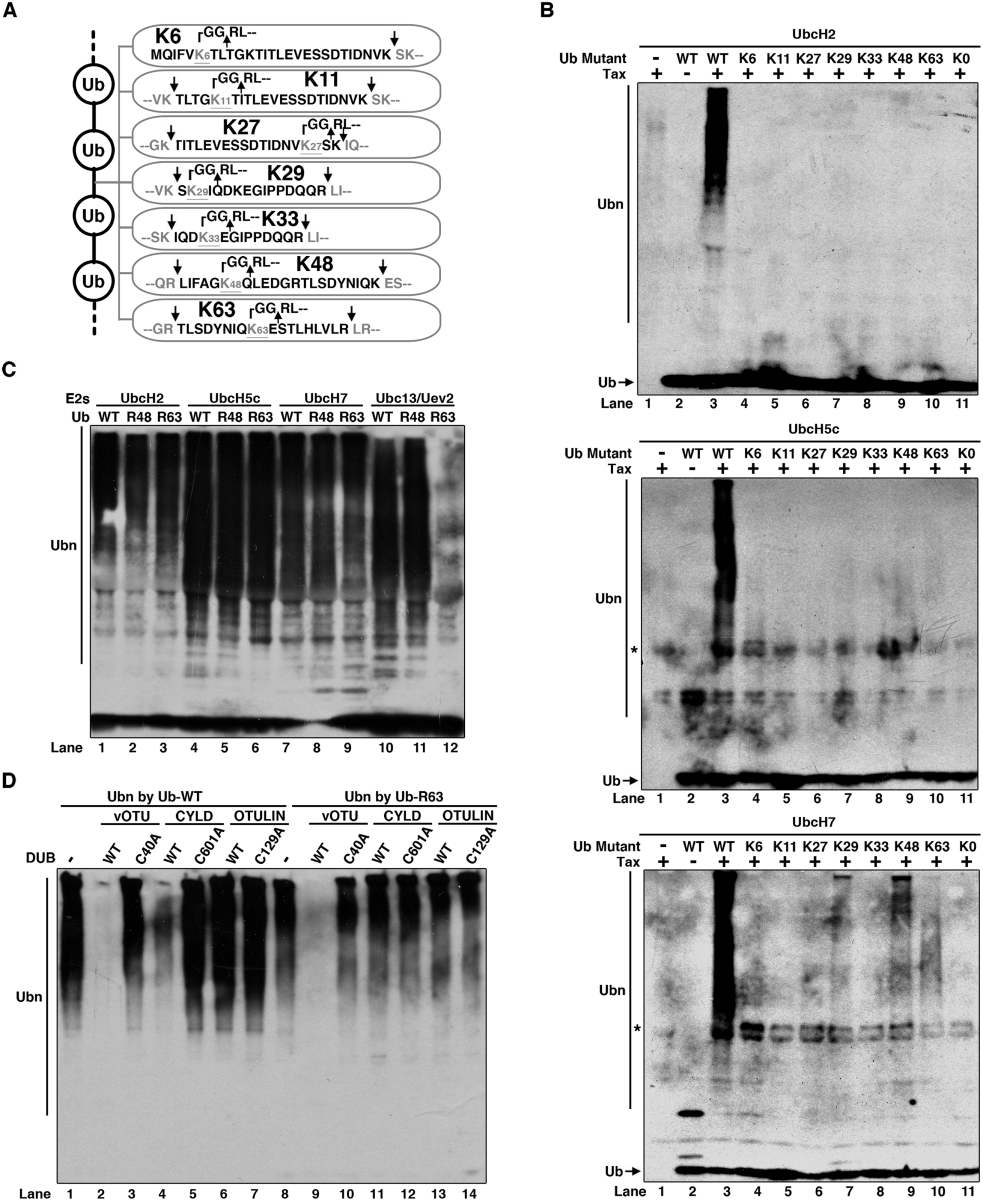
Tax synthesizes free mixed-linkage polyUb chains. **(A)** Diagram showing all the 7 Ub linkages determined by tandem mass spectrometry (MS). **(B)** Polyubiquitin chains synthesized with single K-only Ub mutants. Tax was incubated with E1, the indicated E2s and ubiquitin mutants (diagram was shown in S5B Fig) in ATP buffer at 37°C for 2 h. The products were immunoblotted by using anti-ubiquitin antibody. *: Non-specific detection of Tax by the anti-ubiquitin antibody. **(C)** The same ubiquitination assay as in **(B)** was performed except the ubiquitin was replaced by Ub WT, R48 or R63 as indicated. **(D)** Cleavage of polyUb chains by DUBs. The reaction products in **(C)** by Ub WT or R63 were incubated with DUBs (vOTU, CYLD or OTULIN, or their respective enzymatic-dead mutants) as indicated, at 30°C for 1 h followed by immunoblotting using anti-Ub antibody.

We next tried to characterize what kinds of Ub linkages are formed by Tax. We carried out the ubiquitination reactions by using UbcH7 and Tax or UbcH5c and Tax. The Ub linkages were determined by using mass spectrometric (MS) analysis. As shown in Fig 6A and S5A Fig, all the possible Ub linkages, K6, K11, K27, K29, K33, K48 and K63, were detected, suggesting the polyUb chains are mixed linkage chains. Notably, there was no detectable M1 linkage. To further verify the linkage specificity, we tested a panel of single lysine (K)-only Ub mutants for their ability in polyUb chain synthesis (Fig 6B and S5B–S5D Fig). A single K-only Ub mutant contains only one K with the rest six K residues mutated to arginine (R). Although Ub WT supported the synthesis of polyUb chains, all the other 7 single K-only mutants didn’t show detectable polyUb signals. On the other hand, mutants R48 and R63 (S5B and S5C Fig), each of which has 6 intact K residues, were able to support Tax-dependent synthesis of polyUb chains (Fig 6C). These polyUb chains could be partially digested by CYLD WT but not its enzymatic-dead mutant C601A, suggesting the presence of K63 and/or M1 linkage as well as other linkages in the polyUb chains, which was digested by vOTU WT but not its mutant C40A (Fig 6D). CYLD is a K63- and M1-specific DUB [51]; and vOTU is an ovarian tumor (OTU) domain-containing DUB from Crimean-Congo hemorrhagic fever virus (CCHFV) large (L) protein [52] that can cleave all kinds of Ub linkages except M1 linkage [53]. Since the M1-specific DUB OTULIN didn’t show detectable cleavage on the polyUb chains, which is consistent with the absence of M1 linkage in the polyUb chains analyzed by mass spectrometry (Fig 6A), the linkage cleavage by CYLD was therefore K63 linked. These results further suggest mixed-linkage polyUb chains are synthesized and any single K residue is not sufficient for polyUb chain synthesis by Tax and its cognate E2s.

### Free mixed-linkage polyUb chains synthesized by Tax activate IKK directly

**Fig 7:**
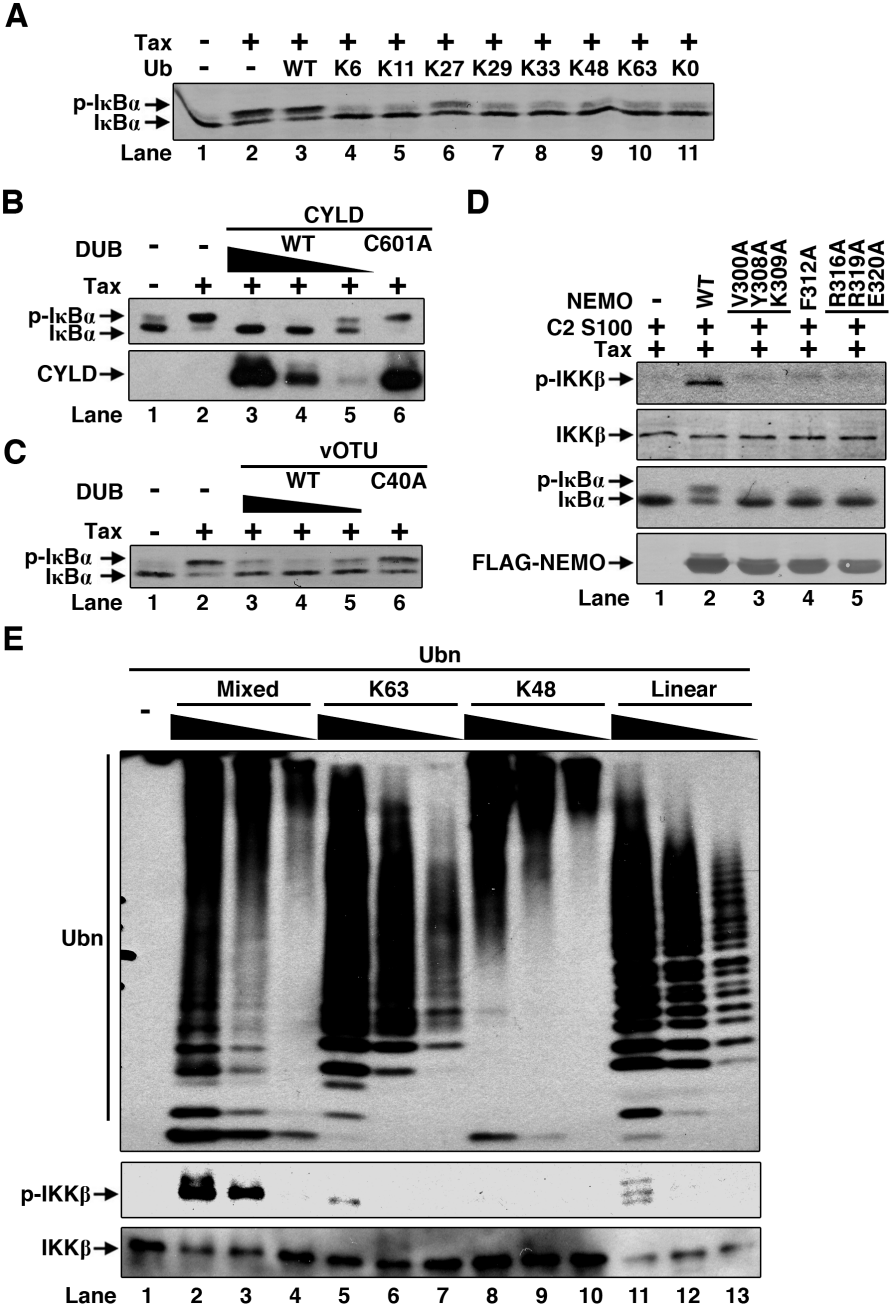
Direct activation of IKK by mixed-linkage polyUb chains synthesized by Tax. **(A)** Ubiquitin mutants impair IKK activation by Tax. Jurkat T S100 was incubated with Tax and the indicated ubiquitin mutants in ATP buffer at 30°C for 1 h. IKK activation was detected by phosphorylation of IκBα. **(B)** Jurkat T S100 was incubated with Tax and different concentrations of CYLD or its enzymatic-dead mutant C601A. IKK activation was detected by phosphorylation of IκBα. **(C)** The same assay as in **(B)** was performed except CYLD was replaced by vOTU or its enzymatic-dead mutant C40A. **(D)** Interaction between NEMO and ubiquitin chains is required for Tax-dependent IKK activation. Cell extracts (S100) of NEMO-deficient Jurkat T (C2) cells were incubated with Tax and NEMO WT or the indicated mutants (100 nM each) in ATP buffer at 30°C for 1 h. IKK activation was detected by phosphorylation of IKKβ and its substrate IκBα. **(E)** Mixed-linkage polyUb chains synthesized by Tax activate IKK directly. Purified IKK complex was incubated with the indicated polyUb chains (prepared and purified as described in Materials and Methods) in ATP buffer at 37°C for 2 h. IKK activation was detected by phosphorylation of IKKβ. Mixed: Mixed linkage polyUb chains synthesized by Tax and UbcH5c. K63: K63 linkage polyUb chains synthesized by TRAF6 and Ubc13/Uev2. K48: K48 linkage polyUb chains synthesized by IpaH and UbcH5c. Linear: Linear linkage polyUb chains synthesized by HOIP-RBRC and UbcH7.

Since Tax catalyzes the synthesis of free mixed-linkage polyUb chains, we wondered if the formation of this kind of mixed-linkage polyUb chains is required for IKK activation by Tax. Again we examined the panel of single K-only Ub mutants for their ability to interfere IKK activation in our S100-based assay system. Addition of Ub WT had no effect on IKK activation by Tax, similar to the one without exogenous Ub. In contrast, inclusion of all the single K-only mutants and the lysine-null (KO) mutant in the reaction didn’t show IKK activation, presumably due to their dominant-negative effect on mixed-linkage polyUb chain assembly and thus inability to support IKK activation by Tax (Fig 7A). Furthermore, addition of viral OTU (vOTU) WT but not the mutant C40A, or CYLD WT but not the mutant C601A, also blocked IKK activation by Tax (Fig 7B and 7C). Taken together, these data further demonstrate that Tax-mediated IKK activation *in vitro* involves the assembly of mixed-linkage polyUb chains.

The NEMO subunit of IKK complex is important in mediating its activation by polyubiquitination events [54]. We therefore tested a panel of NEMO mutants, which have been shown to be differentially defective in binding to various kinds of polyUb chains [55], to examine if they could be defective in IKK activation by Tax. We generated S100 from NEMO-deficient Jurkat T cells and used them for *in vitro* IKK activation assay. As shown in Fig 7D, although NEMO WT restored IKK activation by Tax, the three mutants were not able to achieve it, implying polyUb binding of NEMO is important for IKK activation.

Finally, we tested if the mixed-linkage polyUb chains can activate IKK directly. So we synthesized mixed-linkage polyUb chains first, treated the products with NEM to inactivate all the enzymes (E1, E2s such as UbcH7 or UhcH5c) [53]. Nickel beads were then used to deplete all the ubiquitination components to undetectable level. The remaining free polyUb chains were then concentrated and mixed with purified IKK complex for activation assay. Following the same protocols, we also produced K63, K48 and M1 polyUb chains. As displayed in Fig 7E, the polyUb chains synthesized by Tax-UbcH7 or Tax-UbcH5c activated IKK in a dose-dependent manner. But the K63, K48 and M1 polyUb chains didn’t show any detectable activity on IKK activation. Together, the purified system demonstrates that the mixed-linkage polyUb chains are required and sufficient for direct IKK activation.

### K63-linkage is not required for IKK activation by Tax

**Fig 8:**
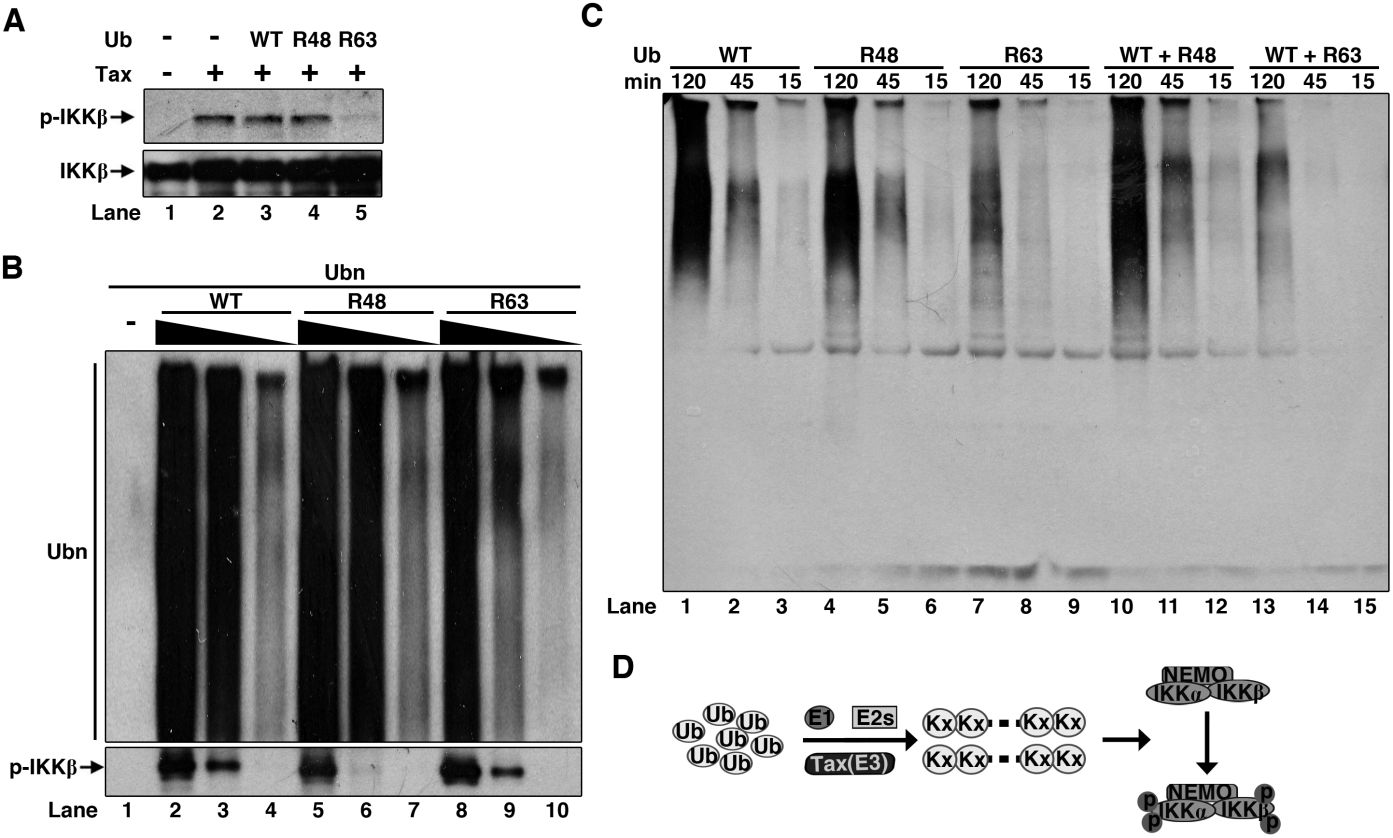
K63 linkage is not required for IKK activation by Tax. **(A)** Ubiquitin R63 mutant impairs IKK activation by Tax. The same assay as in Fig 7A was performed except ubiquitin was replaced by Ub R48 or R63 mutants. **(B)** Polyubiquitin chains pre-synthesized by E1, UbcH5c, Tax and Ub WT or the indicated mutants were incubated with purified IKK complex, in ATP buffer at 37°C for 2 h. IKK activation was detected by phosphorylation of IKKβ. **(C)** Ub R63 is inefficient in polyubiquitination by Tax. Tax was incubated with E1, UbcH5c, and the indicated Ub in ATP buffer at 37°C for the indicated time. Synthesis of polyUb chains was determined by immunoblotting using anti-Ub antibody. **(D)** Model illustrating IKK activation by Tax.

We noted that Tax catalyzed assembly of mixed linkage polyUb chains using Ub mutants R48 and R63 (Fig 6C). So we tested them in the S100-based IKK activation assay. As shown in Fig 8A, both Ub WT and R48 didn’t interfere with Tax-induced IKK activation, but the R63 displayed inhibitory activity, implying the K63 linkage might be a requirement in the mixed linkage polyUb chains for IKK activation. Similar observation was also reported by Shibata et al. [37]. However, polyUb chains assembled from R63 by Tax was able to activate highly purified IKK directly without detectable differences from polyUb chains by either Ub WT or R48 (Fig 8B). Detailed time-course analysis of polyUb chain assembly by Tax revealed the difference of efficiency of polyUb chain assembly among Ub WT, R48 and R63 and could explain the seemingly contradictory results shown in Fig 8A and 8B. Although Ub WT, R48 and R63 all supported polyUb chain assembly, with R63 it displayed slower kinetics and accumulated much less amount of polyUb chains, especially those high molecular size polyUb species. Mixing of Ub WT and R63 together didn’t improve the polyUb chain assembly efficiency (Fig 8C). Together, these data illustrate that K63 linkage in the polyUb chains is not required for IKK activation.

## Discussion

HTLV-1 Tax is the first pathogenic agent that has been shown to be able to activate NF-κB and studies on it have advanced greatly our current understanding of pathophysiological activation of NF-κB. However, despite extensive studies spanning almost 30 years, our understanding of the molecular mechanism by which Tax activates IKK-NF-κB is still far from clear. Like other IKK-NF-κB activation events, Tax-dependent IKK-NF-κB activation induces a variety of cytokines such as TNFα and IL-1β [45], which in turn activate IKK-NF-κB through an autocrine and paracrine fashion, complicating the dissection of signaling events of Tax-dependent IKK activation. We reconstituted the IKK activation events by Tax in an *in vitro* cell-free system. This system captured the primary events of IKK activation induced by Tax. With this system, we identified the essential cellular components required by Tax for IKK activation and revealed the biochemical function of Tax in this process. We found Tax itself is an E3 ubiquitin ligase, which utilizes UbcH7, UbcH2 or UbcH5 as the cognate E2 enzymes to catalyze the assembly of mixed-linkage polyUb chains. This kind of polyUb chains then stimulates activation of IKK directly. This kind of Tax-induced IKK activation is probably the simplest system, which is to a great extent of advantage to HTLV-1 infection by minimizing the events required for IKK-NF-κB activation, an essential event for successful infection of HTLV-1. In other words, HTLV-1 hijacks the minimal host cell components for its own use, minimizing the possible events cells can use for negative regulation of IKK-NF-κB activation and maximizing success of HTLV-1 infection. In the IL-1R/TLR and TNFR signaling pathways, there are multiple steps and key components leading to IKK activation are subject to negative regulation, mainly by DUBs such as A20 [56] and CYLD [57, 58] and protein phosphatases such as PP1 [59] and DUSP14 [60], to subside NF-κB activation. Since Tax-dependent IKK activation is almost at the IKK level and so those negative regulation mechanisms are not effective on Tax-induced IKK activation. This might contribute to Tax-dependent persistent activation of IKK and is beneficial to HTLV-1 infection. Exactly how much this kind of IKK activation contributes to constitutive IKK activation by Tax needs further investigation. Shibata et al. has reported a similar *in vitro* cell-free system to study Tax-dependent IKK activation, in which it reported K63 polyubiquitination is involved in this process. But they didn’t go further to identify essential factors for IKK activation by Tax [37].

There have been reports suggesting both TAK1 and TRAF6 are important for Tax-induced IKK activation. Our *in vitro* fractionation studies unequivocally show that neither TAK1 nor TRAF6 are required for Tax-induced IKK activation. In further support of TAK1- and TRAF6-independent activation of IKK by Tax, we generated TAK1 and TRAF6 KO cells using CRISPR/Cas9 technology. *In vitro* assay with S100 from these KO cells and luciferase reporter assays also demonstrate TAK1 and TRAF6 are not involved in Tax-dependent IKK activation. By using S100 generated from TRAF6 KO MEF cells, Shibata et al. also show TRAF6 is not required for IKK activation by Tax, consistent with our findings [37]. Having said that, however, in cell-based studies, it is highly possible to conclude that TAK1 and TRAF6 are involved in Tax-dependent IKK activation. Cytokines such as IL-1β induced by primary Tax-stimulated IKK-NF-κB activation depend on TAK1 and TRAF6 for IKK activation and there are difficulties to distinguish signaling events by Tax from those autocrine or paracrine signaling events by cytokines.

It is generally accepted that ubiquitination is involved in Tax-induced IKK activation. However, detailed biochemical mechanisms regarding its involvement are not clear, especially about K63 polyubiquitination events. Our studies clarified the confusion and demonstrated that K63 linkage is not a requirement for IKK activation by Tax. We provided several lines of evidence to support this conclusion. In our fractionation experiments, which are an unbiased means for factor identification, Ubc13 was not co-purified with IKK activation activity. Also, inactivation of Ubc13 by OspI in cells and in our S100-based cell-free system didn’t prevent IKK activation from Tax, making it unlikely that K63 polyubiquitination is required for Tax-dependent IKK activation. Together with UbcH7, UbcH2 or UbcH5c, Tax catalyzes assembly of mixed-linkage but not K63 polyUb chain. Although Tax can assemble K63 polyUb chains when the E2 is Ubc13/Uev2, in which the linkage specificity is determined by Ubc13 and Uev2 [61, 62], the K63 polyUb chains are not able to activate IKK. What the function is for K63 polyUb chains catalyzed by Tax is not known yet; it probably functions in DNA damage repair pathway, a reflection of nuclear Tax function [43].

Ub mutant R63 in the S100 inhibited IKK activation by Tax, which would argue for the requirement of K63 linkage for Tax-regulated IKK activation. However, polyUb chains synthesized by Tax using R63 displayed activity on IKK activation, clearly demonstrating that K63 linkage is not a requirement. We were puzzled initially by those seemingly conflicting results. However, our time-course analysis comparing the efficiency of polyUb chain synthesis between Ub WT and R63 provided the answer to the puzzle. Although R63 can support polyUb chain synthesis, its efficiency is low when compared to Ub WT. Mixing of Ub WT and R63 didn’t improve the efficiency of polyUb chain assembly, implying inclusion of R63 in S100 would interfere with polyUb chain assembly leading to insufficiency of polyUb accumulation for IKK activation.

Tax interacts with IKK through the NEMO subunit, which has been suggested to be required for IKK activation by Tax. Tax also undergoes K63 polyubiquitination and induces K63 polyubiquitination of NEMO and these events are also important for IKK activation by Tax [16]. Here, using purified IKK complex and purified polyUb chains synthesized by Tax, we show Tax as well as its ubiquitination is not required in the IKK activation process. Since there are no ubiquitination components in our purified polyUb chains, it is unlikely that NEMO ubiquitination happens during the activation assay in our system. In agreement with our results, Shibata et al. detected Tax-induced NEMO ubiquitination in their system but this event was not required for IKK activation [37]. We conclude Tax interaction with NEMO and NEMO polyubiquitination, if there is any, are not required or at least not critical for Tax-induced IKK activation, clarifying the confusing reports in the literature regarding the molecular mechanism of Tax-dependent IKK activation. However, we cannot exclude that Tax interaction with NEMO and even Tax-induced NEMO polyubiquitination would contribute to IKK activation in cells. We would also stress in cells Tax ubiquitination is still possible which, together with its other PTM modifications such as sumoylation, phosphorylation and acetylation, could regulate some of its other functions such as balancing its cytosol-nucleus translocation [63].

Our studies assigned a novel biochemical function to Tax, that is, as an E3 ubiquitin ligase. It catalyzes assembly of mixed linkage polyUb chains with some of its cognate E2s. Our previous studies have demonstrated that mixed linkage polyUb chains are not able to activate TAK1 complex, which can only be effectively activated by K63 polyUb chains [53]. In the same study we have also shown that mixed-linkage polyUb chains assembled by TRAF6 and UbcH5c were able to activate IKK [53]. In this study, we showed purified free mixed linkage polyUb chains are potent activators towards IKK complex, but other kinds of polyUb chains such as K48, K63 and M1 are poor activators for direct IKK activation, providing another example of direct IKK activation by free mixed polyUb chains. Therefore, we would propose the following model by which Tax activates IKK (Fig 8D): Tax, as a novel E3 ubiquitin ligase, together with its cognate E2s such UbcH7, assembles unanchored free polyUb chains with various linkages, the latter in turn function as potent activators for direct IKK activation. Here mixed linkage is a requirement for efficient IKK activation.

In conclusion, by taking advantage of the *in vitro* cell-free system, we investigated the biochemical mechanism of IKK activation by HTLV-1 encoded Tax protein and the biochemical function of Tax in this process. Tax itself is an E3 ubiquitin ligase, which works together with its cognate E2s, catalyzes free polyUb chains assembly for direct IKK activation. Understanding the biochemical function of Tax for IKK activation and the underlying molecular mechanism clarifies the confusion on HTLV-1-induced IKK-NF-κB activation, reveals the essential cellular factors hijacked by HTLV-1, and might shed light on potential development of therapeutics for ATL and TSP/HAM.

## Materials and Methods

### Antibodies

Rabbit antibodies against IKKα (sc-7218, dilution 1:1,000), IKKβ (sc-7607, dilution 1:1,000), NEMO (sc-8330, dilution 1:1,000) and mouse antibody against ubiquitin (sc-8017, dilution 1:1,000) were obtained from Santa Cruz Biotechnology; antibodies against TAK1 (ab109526, dilution 1:1,000), UbcH7 (ab108936, dilution 1:2,000), Ubc13 (ab109286, dilution 1:5,000), RNF8 (ab131221, dilution 1:1,000), p-IKKα S176 (ab138426, dilution 1:500) were from Abcam; antibodies against CYLD (#8462, dilution 1:1,000), p-TAK1 Thr187 (#4536, dilution 1:1,000), p-IKKα/β Ser176/180 (#2697, dilution 1:1,000) and p-IκBα Ser32/36 (#9246, dilution 1:1,000) were from Cell Signaling; antibodies against FLAG-tag (M20008, dilution 1:5,000) and His-tag (M20001, dilution 1:1,000) were from Abmart; antibodies against IκBα and UbcH5c were homemade; and antibody against Tax (mAB, clone Lt-4) was described in Lee et al. [64].

### Cell culture and transfection

HEK293T and Jurkat T cells were originally from the American Type Culture Collection (ATCC, Manassas, VA). The 293F cells were originally from Thermo Fisher Scientific (New York, NY). The NEMO-deficient Jurkat T cells were kindly provided by Shao-Cong Sun (M. D. Anderson Cancer Center, Houston, TX).

HEK293T and 293F cells were cultured in Dulbecco’s modified Eagle’s medium (DMEM) supplemented with 10% (v/v) fetal bovine serum (Gibco), penicillin (100 U/ml), and streptomycin (100 mg/ml). 293F cells were suspended in SMM 293-T1 medium (Sino Biological Inc.) with antibiotics. Jurkat T and NEMO-deficient Jurkat T cells were cultured in RPMI 1640 supplemented with 10% fetal bovine serum, 2 mM β-mercaptoethanol, penicillin (100 U/ml), and streptomycin (100 mg/ml). Plasmid DNA transfection of 293T and 293F cells was performed using Polyethylenimine (#24765, Polysciences Inc.).

### Generation of knock-out cell lines in 293T cells

Guide RNA sequences (gRNAs) for each gene (TRAF6: 5’-GTCTCCACCCGCTTTGACAT-3’; TAK1: 5’-GCAATGCAAAAAACAACTAG-3’) were cloned into a CRISPR/Cas9-based vector modified from pX330 [65] with a puromycin resistance selection marker. This vector was transfected into 293T cells. After selection by puromycin (0.5 μg/ml), single colonies were picked and verified by western blotting.

### Generation of NEMO-FLAG knock-in cell line in 293F cells

The targeting vector for knock-in contains 1Kb homology arms on each side around the stop codon of human NEMO gene, and a FLAG-IRES-Puromycin segment just before the stop codon. This vector was co-transfected with the CRISPR/Cas9-based vector pX335 [65] (gRNA sequence: 5’-GTCATGGAGTGCATTGAGTA-3’) into 293F cells. After selection by puromycin (0.5 μg/mL), single clones were picked and verified by western blotting.

### Expression constructs and recombinant proteins

cDNAs encoding human UbcH5c (UBE2D3) wild-type (WT) and dominant-negative (DN) mutant (C85A), UbcH7 (UBE2L3) WT and DN mutant (C86A) and Ubc13 (UBE2N) were inserted into plasmids pET15b, pET16b and pET14b, respectively. cDNAs encoding human UEV2 (UBE2V2), UBE2G2, Cdc34 (UBE2R1), UBE2T and CCHFV OTU (1–169) were inserted into plasmid pGEX-4T-1. cDNAs encoding human UBE2A, UBE2B, UbcH10 (UBE2C), UbcH5a (UBE2D1), UbcH5b (UBE2D2), UbcH5d (UBE2D4), UbcH6 (UBE2E1), UBE2G1, UbcH2 (UBE2H), UBE2R2 and human HOIP_697-1072_ (HOIP-RBRC) were inserted into a modified pGEX-4T-1 vector in which the GST tag was replaced by a MBP-His_10_ tag. cDNAs encoding *Shigella* OspI and IpaH_265-568_ were inserted into plasmid pGEX-6P-2. cDNA encoding ubiquitin was inserted into a modified pET-14b vector in which no tag was expressed. cDNAs encoding human E1 and mouse TRAF6 were inserted into pFastBac-HTB vector, cDNAs encoding HTLV-1 Tax WT and M22 (T130AL131S) mutant were inserted into a modified pFastBac-HTB vector in which a MBP tag was inserted into the N-terminus, and a FLAG-His_10_ (10xHis) tag the C-terminus. His_6_-tagged E1, TRAF6 and MBP-Tax-FLAG-His_10_ were expressed in Sf9 cells. cDNAs encoding HTLV-1 Tax WT and M22 mutant were also inserted into a modified pcDNA3.1 vector for mammalian cell expression in which a MBP tag was inserted into the N-terminus, and a FLAG-His_10_ (10xHis) tag the C-terminus. cDNAs encoding human IκBα, NEMO, CYLD, OTULIN, UbcH5c DN, UbcH7 DN and UbcH2 DN (C87A) were inserted into pcDNA3.1-FLAG-His_10_ vector. cDNA for mouse TRAF6 was cloned into pcDNA3.1 with an N-terminal FLAG tag.

The *E*.*coli* strain BL21(DE3/pLys) harboring plasmids of E2s and ubiquitin were induced with 0.5 mM IPTG at 37°C for 4 h, and those harboring the other plasmids were induced with 0.1 mM IPTG at 16°C overnight. His-tagged proteins were purified using nickel agarose beads according to the manufacture’s protocol (Thermo, #88223). FLAG-tagged proteins were transiently expressed in 293T cells and purified using anti-FLAG M2 magnetic beads according to the manufacturer's instructions (Sigma, M8823).

Protein purity was shown in S1, S3A, S3C, S4A and S5C Figs.

### Preparation of cytosolic extract (S100 or S20)

Cell pellet was collected and resuspended in equal volume of hypotonic buffer [20 mM HEPES-KOH, pH 7.4, 10 mM KCl, 1.5 mM MgCl_2_, 1 mM EDTA, 1 mM EGTA, 1 mM DTT, 1 mM PMSF], then homogenized using a Dounce homogenizer. After the cell debris was removed by centrifugation at 20,000×g for 30 min, the supernatant (S20) was collected and stored at -80°C, or further undergone ultracentrifugation at 100,000×g for 1 h and the cleared supernatant (S100) was collected and stored at -80°C.

### Reporter assay for NF-κB activity

Plasmids encoding indicated inhibitory proteins (OspI or dominant-negative mutants of E2s) were transfected into 293T cells first. After 6 hours, cells were transfected again with plasmids encoding Tax or TRAF6, together with a NF-κB firefly-luciferase reporter (3xκB-Luc) and a renilla-luciferase as the internal reference. A pcDNA3.1 empty vector was used to bring total DNA in each transfection group equal. After 24 hours, cells were harvested and luciferase activities were measured.

### *In vitro* cell-free IKK activation assay

To measure the activation of IKK by Tax *in vitro*, cell extracts (S100) of Jurkat T or NEMO-deficient Jurkat T cells were incubated with recombinant Tax, M22 or TRAF6 (100 nM each), and NEMO (100 nM), DUBs or OspI (100 nM) (as shown in figures) in ATP buffer [50 mM Tris-Cl (pH 7.5), 5 mM MgCl_2_, 2 mM ATP, 0.1 μM okadaic acid, 0.5 mM DTT]. After incubation at 30°C for 1 h, the reaction products were immunoblotted using antibodies as specified in each figure. To follow the activity of IKK activation during fractionation, the same IKK assay was performed except that S100 was replaced by Q/III, E1 (20 nM), Ub (10 μM), and aliquots from column fractions. For E2 screening, the same assay was used except that column fractions were replaced by recombinant E2s (0.5 μM).

### Fractionation and purification of UbcH7

All procedures were carried out at 4°C. Jurkat T S100 from 20 L of suspension culture were prepared and applied to HiTrap Q column with buffer Q/A [20 mM HEPES-KOH, pH 7.4, 10% Glycerol, 1 mM EDTA, 1 mM EGTA, 1 mM DTT, 1 mM PMSF], which was eluted with Q/B [Q/A with 1 M NaCl] to generate fractions Q/I, Q/II and Q/III. Q/I was subjected to ammonium sulfate precipitation (40%–80%), followed by dialysis against Buffer SP/A [20 mM HEPES-KOH, pH 6.5, 10% Glycerol, 1 mM EDTA, 1 mM EGTA, 1 mM DTT, 1 mM PMSF]. The dialyzed proteins were applied to HiTrap SP column with buffer SP/A and SP/B [SP/Q with 1 M NaCl]. Fractions containing the activity were pooled and buffer exchanged by ultrafiltration into buffer Q/A, and applied to HiTrap Heparin column with buffer Q/A and Q/B. Fractions that contained the activity were pooled and concentrated before loading onto a Superdex75 column, which was pre-equilibrated with buffer Q/C [Q/A with 100 mM NaCl].

### Ubiquitin charging and discharging assays

To load ubiquitin to recombinant E2s, each E2 (5 μM) was incubated with E1 (100 nM) and Ub (50 μM) in ATP buffer at 30°C for 10 min. The reaction products were resolved by non-reducing SDS-PAGE and E2~Ub intermediate was detected by Coomassie brilliant blue staining. To discharge Ub from E2s by Tax, the same Ub loading assay was used to prepare E2~Ub intermediate. After desalting by G-25 (GE Healthcare) to remove ATP, the intermediates were incubated with recombinant Tax (1 μM) at 30°C for 15 min before immunoblotting.

### Polyubiquitination assay and polyUb chain purification

To synthesize polyUb chains by Tax, recombinant E1 (20 nM), Ub (50 μM), E2 (500 nM) and Tax (1 μM) were incubated in ATP buffer in a final volume of 10 μl at 37°C for 2 h, and the reaction products were used for immunoblotting.

For further purification of polyUb chains by Tax used in Figs 7E and 8B, the reaction volume was scaled up and the final products were mixed with equal volume of Ni-NTA beads for 2 hours at 4°C to deplete His-tagged E1, E2 and Tax. The supernatant was then treated with 10 mM N-ethylmaleimide (NEM) to inactivate any possible remaining E1 and E2, and then NEM was quenched by DTT and removed by ultrafiltration. PolyUb chains in the supernatant were concentrated by ultrafiltration (Millipore, UFC500396).

To purify K48-linkage polyUb chains, similar procedure was carried out except that E3 was replaced with IpaH (10 nM). For K63-linkage polyUb chains, similar procedure was carried out except that E2 and E3 were replaced with Ubc13/Uev2 (1 μM) and TRAF6 (100 nM), respectively. For Linear-linkage (M1) polyUb chains, similar procedure was carried out except that E2 and E3 were replaced with UbcH7 (1 μM) and HOIP-RBRC (250 nM), respectively.

### Reconstitution of IKK activation assay

To reconstitute IKK activation by E2s and Tax, 0.5 ng/μL IKK complex purified from 293F/NEMO-FLAG knock-in cells was incubated with recombinant E1 (20 nM), Ub (10 μM), Tax (300 nM) and the indicated E2 (50 nM) in ATP buffer in a final volume of 10 μl at 37°C for 2 h. To reconstitute IKK activation by polyUb chains, IKK complex (0.5 ng/μL, final volume 10 μl) was incubated with purified polyUb chains in ATP buffer at 37°C for 2 h. Activation of IKK was determined by immunoblotting using phospho-IKKα/β antibody.

### In solution tryptic digestion of polyUb chains

The proteins were precipitated by TCA and then tryptically digested following the procedure described previously [66]. Briefly, the protein precipitate were resolved by 8 M Urea, and then sequentially treated with 5 mM TCEP and 10 mM NEM to reduce the di-sulfide bond and alkylate the resulting thiol group. The mixture was digested for 16 h at 37°C by trypsin at an enzyme-to-substrate ratio of 1:50 (w/w).

### HPLC-MS/MS

The trypsin-digested peptides were desalted with C18 Zip-Tips and then loaded onto an in-house packed capillary reverse-phase C18 column (15 cm length, 100 μM ID x 360 μM OD, 3 μM particle size, 100 Å pore diameter) connected to a Thermo Easy-nLC1000 HPLC system. The samples were analyzed with a 180 min-HPLC gradient from 0% to 100% of buffer B (buffer A: 0.1% formic acid in Water; buffer B: 0.1% formic acid in 20/80 water/acetonitrile) at 300 nL/min. The eluted peptides were ionized and directly introduced into a Q-Exactive mass spectrometer using a nano-spray source. Survey full-scan MS spectra (from *m*/*z* 300–1800) were acquired in the Orbitrap analyzer with resolution *r* = 70,000 at *m*/*z* 400.

### Analysis of tandem mass spectra

Protein identification and post-translational modification analysis were done with Integrated Proteomics Pipeline—IP2 (Integrated Proteomics Applications, Inc., http://www.integratedproteomics.com) using ProLuCID/Sequest [67, 68] and DTASelect2 [69, 70]. Spectrum raw files were extracted into ms2 files from raw files using RawExtract [71], and the tandem mass spectra were searched against the Uniprot human protein database; plus sequences of known contaminants such as keratin and porcine trypsin concatenated to a decoy database in which the sequence for each entry in the original database was reversed using ProLuCID/Sequest. Alkylation (+125.0477) of cysteine and oxidation (+15.9949) of methionine were considered as static modifications, and ubiquitination (+114.0429) was considered as a variable modification. We require 2 peptides per protein and at least one tryptic terminus for each peptide identification. Search space included all fully- and half-tryptic peptide candidates with missed cleavage restrictions.

### Accession numbers

The Uniprot entry IDs for each genes described in the text are: P03409 (Tax), P22314 (E1), P0CG48 (Ubiquitin), P68036 (UbcH7), P62256 (UbcH2), P61077 (UbcH5c), P61088 (Ubc13), Q15819 (Uev2), O15111 (IKKα), O14920 (IKKβ), Q9Y6K9 (NEMO), P25963 (IκBα), O43318 (TAK1), Q8VSD5 (OspI), Q6TQR6 (vOTU), Q96BN8 (OTULIN), Q9NQC7 (CYLD), Q9Y4K3 (TRAF6).

## Supporting Information

S1 Fig Recombinant proteins used in the study. Proteins were purified as described in Materials and Methods. An aliquot of the purified proteins were applied to SDS-PAGE, followed by colloidal blue staining to show their purity.
(TIF)

S2 Fig Fractionation of Jurkat T S100. **(A)** *In vitro* IKK activation assay performed with HiTrap Q fractions. Fractions from Q column (detailed scheme was shown in Fig 2A, left panel) were combined and incubated with Tax (100 nM) and ATP for 1 h at 30°C. IKK activation was detected by phosphorylation of IKKα, IKKβ and their substrate IκBα. IKKα, IKKβ, NEMO, Ubc13, UbcH7 and RNF8 were detected by immunoblotting. **(B)** IKK activation assay throughout the purification. Fractions from SP column (upper panel) and Heparin column (lower panel) of the purification steps (detailed scheme in Fig 2B, left panel) were incubated with Q/III to perform the *in vitro* assay as in **(A)** to follow the activity. SP fractions 20–24, and Heparin fractions 16–18, were used for the next step purification. **(C)** Coomassie brilliant blue staining of the purified recombinant UbcH7 WT and C86A proteins. **(D)** Ubiquitin loading assay to verify UbcH7 activity. Recombinant UbcH7 was incubated with E1 and Ubiquitin in the absence or presence of ATP at 30°C for 10 min. The UbcH7~Ub thioester intermediate was detected by Coomassie brilliant blue staining after separation using 15% non-reducing SDS-PAGE.
(TIF)

S3 Fig Multiple E2s confer Tax-dependent IKK activation. **(A)** Ubiquitin loading assay to verify E2 activity. The same ubiquitin loading assay as in S2D Fig was performed except UbcH7 was replaced with the indicated recombinant E2s. **(B)** Screening of E2s for Tax-dependent IKK activation *in vitro*. The same *in vitro* assay as in Fig 2D was performed except UbcH7 was replaced with the indicated recombinant E2s. **(C)** OspI doesn’t impair IKK activation by Tax *in vitro*. Jurkat T S100 was incubated with Tax WT, M22 or TRAF6, together with OspI WT or its enzymatic-dead mutant (C62A), in ATP buffer at 30°C for 1 h. Phosphorylation of TAK1, IKKβ and its substrate IκBα was detected by immunoblotting. **(D)** OspI doesn’t impair IKK activation by Tax in cells. 293T cells were transfected with OspI WT, C62A, or GFP as a control. After 6 hours, cells were transfected again with plasmids encoding Tax WT, M22 or TRAF6, together with a NF-κB firefly-luciferase reporter and a renilla-luciferase internal control. The luciferase activity was measured and normalized by renilla luciferase. **(E)** TAK1 is not involved in Tax-dependent IKK activation *in vitro*. Cell extracts (S20) of 293T or TAK1-deficient 293T cells were incubated with Tax WT or TRAF6, and ATP at 30°C for 1 h. Phosphorylation of TAK1, IKKβ and its substrate IκBα was detected by immunoblotting. **(F)** TAK1 and TRAF6 are not involved in Tax-dependent IKK activation in cells. 293T WT, TAK1 KO or TRAF6 KO cells were transfected with plasmids encoding Tax WT, M22, TRAF6, IKKβ or GFP, together with a NF-κB firefly-luciferase reporter. The luciferase activity was measured 24 h later and normalized by renilla luciferase. FH: FLAG-10xHis tag.
(TIF)

S4 Fig Direct IKK activation by Tax *in vitro*. **(A)** Silver staining and immunoblotting of IKK complex purified as described in Materials and Methods. **(B)** Ubc13/Uev2 doesn’t support Tax-dependent IKK activation. The same assay as in Fig 4A was performed except UbcH7 was replaced by Ubc13/Uev2.
(TIF)

S5 Fig Assembly of mixed linkage polyUb chains by Tax. **(A)** Identification of Ub linkage assembled by Tax by using Thermo Q-exactive. A brief diagram was shown in Fig 6A. **(B)** Diagram of Ub mutants. **(C)** Colloidal blue staining of recombinant ubiquitin WT or mutant proteins. **(D)** Tax synthesizes K63-linkage polyUb chains with Ubc13/Uev2. The same assay as in Fig 6B was performed except Ubc13/Uev2 was used as the E2.
(TIF)

## References

[1] Poiesz BJ, Ruscetti FW, Gazdar AF, Bunn PA, Minna JD, Gallo RC. Detection and isolation of type C retrovirus particles from fresh and cultured lymphocytes of a patient with cutaneous T-cell lymphoma. Proceedings of the National Academy of Sciences of the United States of America. 1980;77(12):7415–9. 6261256 10.1073/pnas.77.12.7415 PMC350514

[2] Gessain A, Francis H, Sonan T, Giordano C, Akani F, Piquemal M, et al HTLV-I and tropical spastic paraparesis in Africa. Lancet. 1986;2(8508):698 . 2876179 10.1016/s0140-6736(86)90218-7

[3] Matsuoka M, Jeang K-T. Human T-cell leukaemia virus type 1 (HTLV-1) infectivity and cellular transformation. Nature reviews Cancer. 2007;7(4):270–80. Epub 2007/03/27. 10.1038/nrc2111 . 17384582

[4] Giam C-Z, Jeang K-T. HTLV-1 Tax and adult T-cell leukemia. Frontiers in bioscience: a journal and virtual library. 2007;12:1496–507. Epub 2006/11/28. . 17127397 10.2741/2163

[5] Tanaka A, Takahashi C, Yamaoka S, Nosaka T, Maki M, Hatanaka M. Oncogenic transformation by the tax gene of human T-cell leukemia virus type I in vitro. Proceedings of the National Academy of Sciences of the United States of America. 1990;87(3):1071–5. 2300570 10.1073/pnas.87.3.1071 PMC53412

[6] Chan JK, Greene WC. Dynamic roles for NF-kappaB in HTLV-I and HIV-1 retroviral pathogenesis. Immunological reviews. 2012;246(1):286–310. Epub 2012/03/23. 10.1111/j.1600-065X.2012.01094.x . 22435562

[7] Zimmerman B, Niewiesk S, Lairmore MD. Mouse models of human T lymphotropic virus type-1-associated adult T-cell leukemia/lymphoma. Veterinary pathology. 2010;47(4):677–89. 10.1177/0300985810370009 20442421 PMC3147149

[8] Hasegawa H, Sawa H, Lewis MJ, Orba Y, Sheehy N, Yamamoto Y, et al Thymus-derived leukemia-lymphoma in mice transgenic for the Tax gene of human T-lymphotropic virus type I. Nature medicine. 2006;12(4):466–72. 10.1038/nm1389 . 16550188

[9] Shirinian M, Kambris Z, Hamadeh L, Grabbe C, Journo C, Mahieux R, et al A Transgenic Drosophila melanogaster Model To Study Human T-Lymphotropic Virus Oncoprotein Tax-1-Driven Transformation In Vivo. Journal of virology. 2015;89(15):8092–5. 10.1128/JVI.00918-15 25995252 PMC4505646

[10] Yamaoka S, Tobe T, Hatanaka M. Tax protein of human T-cell leukemia virus type I is required for maintenance of the transformed phenotype. Oncogene. 1992;7(3):433–7. . 1549359

[11] Robek MD, Ratner L. Immortalization of CD4(+) and CD8(+) T lymphocytes by human T-cell leukemia virus type 1 Tax mutants expressed in a functional molecular clone. Journal of virology. 1999;73(6):4856–65. 10233947 10.1128/jvi.73.6.4856-4865.1999 PMC112529

[12] Kwon H, Ogle L, Benitez B, Bohuslav J, Montano M, Felsher DW, et al Lethal cutaneous disease in transgenic mice conditionally expressing type I human T cell leukemia virus Tax. The Journal of biological chemistry. 2005;280(42):35713–22. 10.1074/jbc.M504848200 . 16105841

[13] Zhao T, Matsuoka M. HBZ and its roles in HTLV-1 oncogenesis. Frontiers in microbiology. 2012;3:247 Epub 2012/07/13. 10.3389/fmicb.2012.00247 22787458 PMC3391691

[14] Shirinian M, Kfoury Y, Dassouki Z, El-Hajj H, Bazarbachi A. Tax-1 and Tax-2 similarities and differences: focus on post-translational modifications and NF-kappaB activation. Front Microbiol. 2013;4:231 Epub 2013/08/24. 10.3389/fmicb.2013.00231 ; PubMed Central PMCID: PMCPmc3744011. 23966989 PMC3744011

[15] Simonis N, Rual JF, Lemmens I, Boxus M, Hirozane-Kishikawa T, Gatot JS, et al Host-pathogen interactome mapping for HTLV-1 and -2 retroviruses. Retrovirology. 2012;9:26 Epub 2012/03/31. 10.1186/1742-4690-9-26 ; PubMed Central PMCID: PMCPmc3351729. 22458338 PMC3351729

[16] Currer R, Van Duyne R, Jaworski E, Guendel I, Sampey G, Das R, et al HTLV tax: a fascinating multifunctional co-regulator of viral and cellular pathways. Front Microbiol. 2012;3:406 10.3389/fmicb.2012.00406 23226145 PMC3510432

[17] Sun SC, Yamaoka S. Activation of NF-kappaB by HTLV-I and implications for cell transformation. Oncogene. 2005;24(39):5952–64. 10.1038/sj.onc.1208969 . 16155602

[18] Qu Z, Xiao G. Human T-cell lymphotropic virus: a model of NF-κB-associated tumorigenesis. Viruses. 2011;3(6):714–49. Epub 2011/07/12. 10.3390/v3060714 21743832 PMC3131208

[19] Hayden MS, Ghosh S. NF-kappaB, the first quarter-century: remarkable progress and outstanding questions. Genes & development. 2012;26(3):203–34. 10.1101/gad.183434.111 22302935 PMC3278889

[20] Liu F, Xia Y, Parker AS, Verma IM. IKK biology. Immunological reviews. 2012;246(1):239–53. 10.1111/j.1600-065X.2012.01107.x 22435559 PMC3311052

[21] Chen ZJ. Ubiquitination in signaling to and activation of IKK. Immunological reviews. 2012;246(1):95–106. Epub 2012/03/23. 10.1111/j.1600-065X.2012.01108.x . 22435549 PMC3549672

[22] Dai L, Aye Thu C, Liu XY, Xi J, Cheung PC. TAK1, more than just innate immunity. IUBMB life. 2012;64(10):825–34. 10.1002/iub.1078 . 22941947

[23] Chu ZL, Shin YA, Yang JM, DiDonato JA, Ballard DW. IKKgamma mediates the interaction of cellular IkappaB kinases with the tax transforming protein of human T cell leukemia virus type 1. The Journal of biological chemistry. 1999;274(22):15297–300. . 10336413 10.1074/jbc.274.22.15297

[24] Harhaj EW, Sun SC. IKKgamma serves as a docking subunit of the IkappaB kinase (IKK) and mediates interaction of IKK with the human T-cell leukemia virus Tax protein. The Journal of biological chemistry. 1999;274(33):22911–4. . 10438454 10.1074/jbc.274.33.22911

[25] Xiao G, Harhaj EW, Sun SC. Domain-specific interaction with the I kappa B kinase (IKK)regulatory subunit IKK gamma is an essential step in tax-mediated activation of IKK. The Journal of biological chemistry. 2000;275(44):34060–7. 10.1074/jbc.M002970200 . 10906125

[26] Huang GJ, Zhang ZQ, Jin DY. Stimulation of IKK-gamma oligomerization by the human T-cell leukemia virus oncoprotein Tax. FEBS letters. 2002;531(3):494–8. . 12435599 10.1016/s0014-5793(02)03590-1

[27] Xiao G, Sun SC. Activation of IKKalpha and IKKbeta through their fusion with HTLV-I tax protein. Oncogene. 2000;19(45):5198–203. 10.1038/sj.onc.1203894 . 11064457

[28] Huang J, Ren T, Guan H, Jiang Y, Cheng H. HTLV-1 Tax is a critical lipid raft modulator that hijacks IkappaB kinases to the microdomains for persistent activation of NF-kappaB. The Journal of biological chemistry. 2009;284(10):6208–17. 10.1074/jbc.M806390200 . 19129196

[29] Pujari R, Hunte R, Thomas R, van der Weyden L, Rauch D, Ratner L, et al Human T-cell leukemia virus type 1 (HTLV-1) tax requires CADM1/TSLC1 for inactivation of the NF-kappaB inhibitor A20 and constitutive NF-kappaB signaling. PLoS pathogens. 2015;11(3):e1004721 10.1371/journal.ppat.1004721 25774694 PMC4361615

[30] Wu X, Sun SC. Retroviral oncoprotein Tax deregulates NF-kappaB by activating Tak1 and mediating the physical association of Tak1-IKK. EMBO reports. 2007;8(5):510–5. 10.1038/sj.embor.7400931 17363973 PMC1866198

[31] Yin MJ, Christerson LB, Yamamoto Y, Kwak YT, Xu S, Mercurio F, et al HTLV-I Tax protein binds to MEKK1 to stimulate IkappaB kinase activity and NF-kappaB activation. Cell. 1998;93(5):875–84. . 9630230 10.1016/s0092-8674(00)81447-6

[32] Uhlik M, Good L, Xiao G, Harhaj EW, Zandi E, Karin M, et al NF-kappaB-inducing kinase and IkappaB kinase participate in human T-cell leukemia virus I Tax-mediated NF-kappaB activation. The Journal of biological chemistry. 1998;273(33):21132–6. . 9694868 10.1074/jbc.273.33.21132

[33] Babu G, Waterfield M, Chang M, Wu X, Sun SC. Deregulated activation of oncoprotein kinase Tpl2/Cot in HTLV-I-transformed T cells. The Journal of biological chemistry. 2006;281(20):14041–7. 10.1074/jbc.M512375200 . 16565081

[34] Ho YK, Zhi H, Bowlin T, Dorjbal B, Philip S, Zahoor MA, et al HTLV-1 Tax Stimulates Ubiquitin E3 Ligase, Ring Finger Protein 8, to Assemble Lysine 63-Linked Polyubiquitin Chains for TAK1 and IKK Activation. PLoS pathogens. 2015;11(8):e1005102 10.1371/journal.ppat.1005102 . 26285145 PMC4540474

[35] Gohda J, Irisawa M, Tanaka Y, Sato S, Ohtani K, Fujisawa J, et al HTLV-1 Tax-induced NFkappaB activation is independent of Lys-63-linked-type polyubiquitination. Biochemical and biophysical research communications. 2007;357(1):225–30. Epub 2007/04/10. 10.1016/j.bbrc.2007.03.125 . 17418100

[36] Suzuki S, Zhou Y, Refaat A, Takasaki I, Koizumi K, Yamaoka S, et al Human T cell lymphotropic virus 1 manipulates interferon regulatory signals by controlling the TAK1-IRF3 and IRF4 pathways. The Journal of biological chemistry. 2010;285(7):4441–6. 10.1074/jbc.M109.031476 19955181 PMC2836049

[37] Shibata Y, Tanaka Y, Gohda J, Inoue J. Activation of the IkappaB kinase complex by HTLV-1 Tax requires cytosolic factors involved in Tax-induced polyubiquitination. Journal of biochemistry. 2011;150(6):679–86. 10.1093/jb/mvr106 . 21862596

[38] Shembade N, Harhaj NS, Yamamoto M, Akira S, Harhaj EW. The human T-cell leukemia virus type 1 Tax oncoprotein requires the ubiquitin-conjugating enzyme Ubc13 for NF-kappaB activation. Journal of virology. 2007;81(24):13735–42. 10.1128/JVI.01790-07 17942533 PMC2168884

[39] Kfoury Y, Nasr R, Favre-Bonvin A, El-Sabban M, Renault N, Giron ML, et al Ubiquitylated Tax targets and binds the IKK signalosome at the centrosome. Oncogene. 2008;27(12):1665–76. 10.1038/sj.onc.1210804 . 17891179

[40] Carter RS, Pennington KN, Arrate P, Oltz EM, Ballard DW. Site-specific monoubiquitination of IkappaB kinase IKKbeta regulates its phosphorylation and persistent activation. The Journal of biological chemistry. 2005;280(52):43272–9. 10.1074/jbc.M508656200 . 16267042

[41] Yasunaga J, Lin FC, Lu X, Jeang KT. Ubiquitin-specific peptidase 20 targets TRAF6 and human T cell leukemia virus type 1 tax to negatively regulate NF-kappaB signaling. Journal of virology. 2011;85(13):6212–9. 10.1128/JVI.00079-11 21525354 PMC3126525

[42] Lamsoul I, Lodewick J, Lebrun S, Brasseur R, Burny A, Gaynor RB, et al Exclusive ubiquitination and sumoylation on overlapping lysine residues mediate NF-kappaB activation by the human T-cell leukemia virus tax oncoprotein. Molecular and cellular biology. 2005;25(23):10391–406. 10.1128/MCB.25.23.10391-10406.2005 16287853 PMC1291224

[43] Nasr R, Chiari E, El-Sabban M, Mahieux R, Kfoury Y, Abdulhay M, et al Tax ubiquitylation and sumoylation control critical cytoplasmic and nuclear steps of NF-kappaB activation. Blood. 2006;107(10):4021–9. 10.1182/blood-2005-09-3572 . 16424386

[44] Bonnet A, Randrianarison-Huetz V, Nzounza P, Nedelec M, Chazal M, Waast L, et al Low nuclear body formation and tax SUMOylation do not prevent NF-kappaB promoter activation. Retrovirology. 2012;9:77 10.1186/1742-4690-9-77 23009398 PMC3476979

[45] Cowan EP, Alexander RK, Daniel S, Kashanchi F, Brady JN. Induction of tumor necrosis factor alpha in human neuronal cells by extracellular human T-cell lymphotropic virus type 1 Tax. Journal of virology. 1997;71(9):6982–9. 9261427 10.1128/jvi.71.9.6982-6989.1997 PMC191983

[46] Smith MR, Greene WC. Identification of HTLV-I tax trans-activator mutants exhibiting novel transcriptional phenotypes. Genes & development. 1990;4(11):1875–85. . 2276622 10.1101/gad.4.11.1875

[47] Smith M, Greene W. Identification of HTLV-I tax trans-activator mutants exhibiting novel transcriptional phenotypes. Genes & development. 1995;9(18):2324 Epub 1995/09/15. . 2276622 10.1101/gad.4.11.1875

[48] Deng L, Wang C, Spencer E, Yang L, Braun A, You J, et al Activation of the IkappaB kinase complex by TRAF6 requires a dimeric ubiquitin-conjugating enzyme complex and a unique polyubiquitin chain. Cell. 2000;103(2):351–61. . 11057907 10.1016/s0092-8674(00)00126-4

[49] Fu P, Zhang X, Jin M, Xu L, Wang C, Xia Z, et al Complex structure of OspI and Ubc13: the molecular basis of Ubc13 deamidation and convergence of bacterial and host E2 recognition. PLoS pathogens. 2013;9(4):e1003322 10.1371/journal.ppat.1003322 23633953 PMC3636029

[50] Sanada T, Kim M, Mimuro H, Suzuki M, Ogawa M, Oyama A, et al The Shigella flexneri effector OspI deamidates UBC13 to dampen the inflammatory response. Nature. 2012;483(7391):623–6. 10.1038/nature10894 . 22407319

[51] Sato Y, Goto E, Shibata Y, Kubota Y, Yamagata A, Goto-Ito S, et al Structures of CYLD USP with Met1- or Lys63-linked diubiquitin reveal mechanisms for dual specificity. Nature structural & molecular biology. 2015;22(3):222–9. 10.1038/nsmb.2970 . 25686088

[52] Frias-Staheli N, Giannakopoulos NV, Kikkert M, Taylor SL, Bridgen A, Paragas J, et al Ovarian tumor domain-containing viral proteases evade ubiquitin- and ISG15-dependent innate immune responses. Cell host & microbe. 2007;2(6):404–16. 10.1016/j.chom.2007.09.014 18078692 PMC2184509

[53] Xia ZP, Sun L, Chen X, Pineda G, Jiang X, Adhikari A, et al Direct activation of protein kinases by unanchored polyubiquitin chains. Nature. 2009;461(7260):114–9. Epub 2009/08/14. 10.1038/nature08247 19675569 PMC2747300

[54] Clark K, Nanda S, Cohen P. Molecular control of the NEMO family of ubiquitin-binding proteins. Nature reviews Molecular cell biology. 2013;14(10):673–85. 10.1038/nrm3644 . 23989959

[55] Rahighi S, Ikeda F, Kawasaki M, Akutsu M, Suzuki N, Kato R, et al Specific recognition of linear ubiquitin chains by NEMO is important for NF-kappaB activation. Cell. 2009;136(6):1098–109. 10.1016/j.cell.2009.03.007 . 19303852

[56] Shembade N, Ma A, Harhaj EW. Inhibition of NF-kappaB signaling by A20 through disruption of ubiquitin enzyme complexes. Science. 2010;327(5969):1135–9. 10.1126/science.1182364 20185725 PMC3025292

[57] Trompouki E, Hatzivassiliou E, Tsichritzis T, Farmer H, Ashworth A, Mosialos G. CYLD is a deubiquitinating enzyme that negatively regulates NF-kappaB activation by TNFR family members. Nature. 2003;424(6950):793–6. 10.1038/nature01803 . 12917689

[58] Harhaj EW, Dixit VM. Regulation of NF-kappaB by deubiquitinases. Immunological reviews. 2012;246(1):107–24. 10.1111/j.1600-065X.2012.01100.x 22435550 PMC3540820

[59] Gu M, Ouyang C, Lin W, Zhang T, Cao X, Xia Z, et al Phosphatase holoenzyme PP1/GADD34 negatively regulates TLR response by inhibiting TAK1 serine 412 phosphorylation. J Immunol. 2014;192(6):2846–56. Epub 2014/02/19. 10.4049/jimmunol.1302537 . 24534530

[60] Zheng H, Li Q, Chen R, Zhang J, Ran Y, He X, et al The dual-specificity phosphatase DUSP14 negatively regulates tumor necrosis factor- and interleukin-1-induced nuclear factor-kappaB activation by dephosphorylating the protein kinase TAK1. The Journal of biological chemistry. 2013;288(2):819–25. 10.1074/jbc.M112.412643 23229544 PMC3543031

[61] Eddins MJ, Carlile CM, Gomez KM, Pickart CM, Wolberger C. Mms2-Ubc13 covalently bound to ubiquitin reveals the structural basis of linkage-specific polyubiquitin chain formation. Nature structural & molecular biology. 2006;13(10):915–20. Epub 2006/09/19. 10.1038/nsmb1148 . 16980971

[62] VanDemark AP, Hofmann RM, Tsui C, Pickart CM, Wolberger C. Molecular insights into polyubiquitin chain assembly: crystal structure of the Mms2/Ubc13 heterodimer. Cell. 2001;105(6):711–20. Epub 2001/07/07. . 11440714 10.1016/s0092-8674(01)00387-7

[63] Bidoia C. Human T-lymphotropic virus proteins and post-translational modification pathways. World journal of virology. 2012;1(4):115–30. Epub 2012/08/12. 10.5501/wjv.v1.i4.115 ; PubMed Central PMCID: PMCPmc3782272. 24175216 PMC3782272

[64] Lee B, Tanaka Y, Tozawa H. Monoclonal antibody defining tax protein of human T-cell leukemia virus type-I. The Tohoku journal of experimental medicine. 1989;157(1):1–11. . 2711372 10.1620/tjem.157.1

[65] Cong L, Ran FA, Cox D, Lin S, Barretto R, Habib N, et al Multiplex genome engineering using CRISPR/Cas systems. Science. 2013;339(6121):819–23. 10.1126/science.1231143 23287718 PMC3795411

[66] Peng C, Lu Z, Xie Z, Cheng Z, Chen Y, Tan M, et al The first identification of lysine malonylation substrates and its regulatory enzyme. Molecular & cellular proteomics: MCP. 2011;10(12):M111 012658. 10.1074/mcp.M111.012658 21908771 PMC3237090

[67] Eng JK, McCormack AL, Yates JR. An approach to correlate tandem mass spectral data of peptides with amino acid sequences in a protein database. Journal of the American Society for Mass Spectrometry. 1994;5(11):976–89. 10.1016/1044-0305(94)80016-2 . 24226387

[68] Xu T, Park SK, Venable JD, Wohlschlegel JA, Diedrich JK, Cociorva D, et al ProLuCID: An improved SEQUEST-like algorithm with enhanced sensitivity and specificity. J Proteomics. 2015;129:16–24. 10.1016/j.jprot.2015.07.001 26171723 PMC4630125

[69] Cociorva D, T D L, Yates JR. Validation of tandem mass spectrometry database search results using DTASelect. Curr Protoc Bioinformatics. 2007;Chapter 13:Unit 13 4 10.1002/0471250953.bi1304s16 . 18428785

[70] Tabb DL, McDonald WH, Yates JR 3rd. DTASelect and Contrast: tools for assembling and comparing protein identifications from shotgun proteomics. Journal of proteome research. 2002;1(1):21–6. 12643522 10.1021/pr015504q PMC2811961

[71] McDonald WH, Tabb DL, Sadygov RG, MacCoss MJ, Venable J, Graumann J, et al MS1, MS2, and SQT-three unified, compact, and easily parsed file formats for the storage of shotgun proteomic spectra and identifications. Rapid communications in mass spectrometry: RCM. 2004;18(18):2162–8. 10.1002/rcm.1603 . 15317041

