# Supplementary figures and images for: HTLV-1 Tax Functions as a Ubiquitin E3 Ligase for Direct IKK Activation via Synthesis of Mixed-Linkage Polyubiquitin Chains
Source: PLoS Pathog. 2016 Apr 15;12(4):e1005584. doi: 10.1371/journal.ppat.1005584 (PMC4833305; doi:10.1371/journal.ppat.1005584)

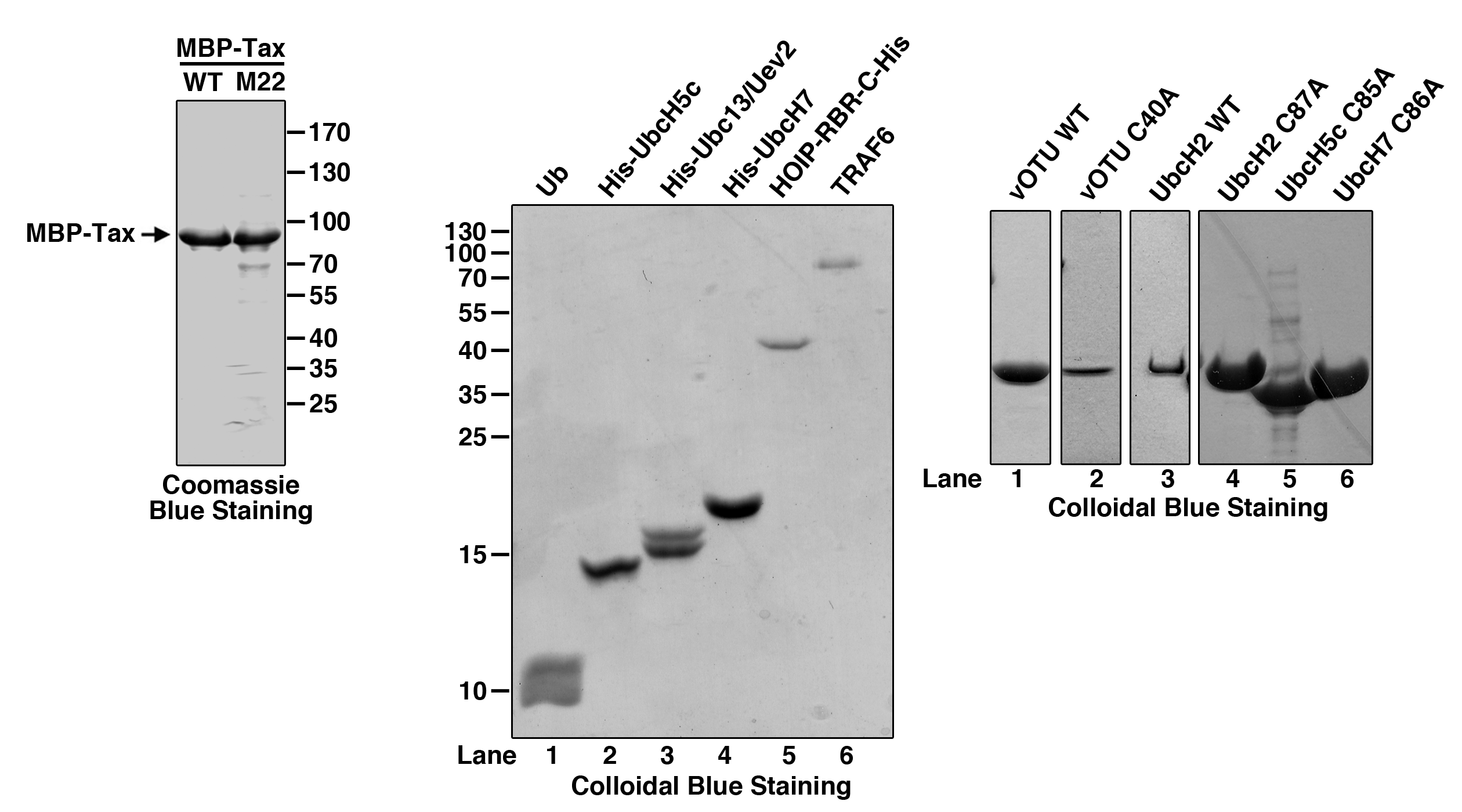

Supplement: S1 Fig — Proteins were purified as described in Materials and Methods. An aliquot of the purified proteins were applied to SDS-PAGE, followed by colloidal blue staining to show their purity. (TIF) [file ppat.1005584.s001.tif]

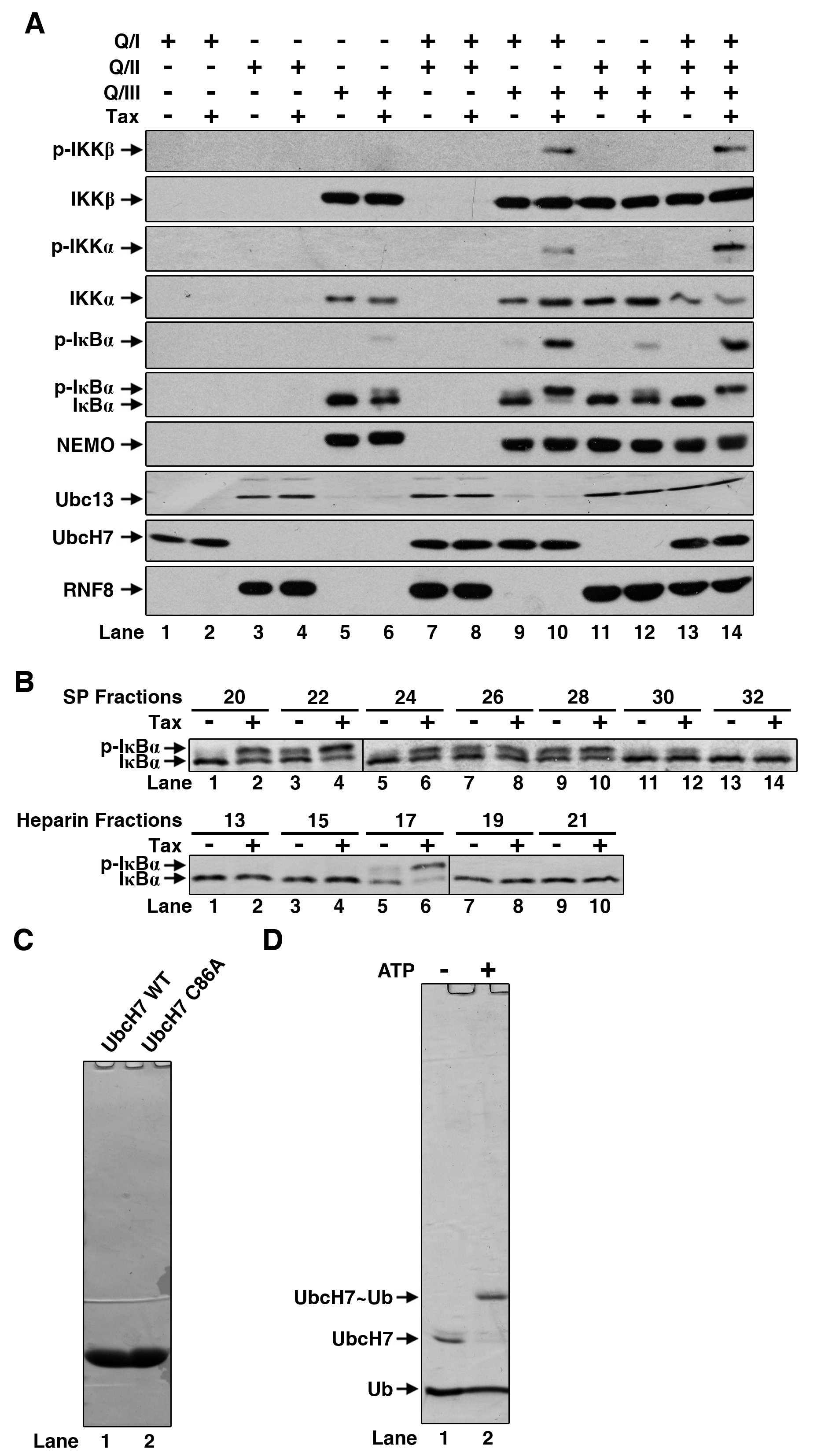

Supplement: S2 Fig — (A) In vitro IKK activation assay performed with HiTrap Q fractions. Fractions from Q column (detailed scheme was shown in Fig 2A, left panel) were combined and incubated with Tax (100 nM) and ATP for 1 h at 30°C. IKK activation was detected by phosphorylation of IKKα, IKKβ and their substrate IκBα. IKKα, IKKβ, NEMO, Ubc13, UbcH7 and RNF8 were detected by immunoblotting. (B) IKK activation assay throughout the purification. Fractions from SP column (upper panel) and Heparin column (lower panel) of the purification steps (detailed scheme in Fig 2B, left panel) were incubated with Q/III to perform the in vitro assay as in (A) to follow the activity. SP fractions 20–24, and Heparin fractions 16–18, were used for the next step purification. (C) Coomassie brilliant blue staining of the purified recombinant UbcH7 WT and C86A proteins. (D) Ubiquitin loading assay to verify UbcH7 activity. Recombinant UbcH7 was incubated with E1 and Ubiquitin in the absence or presence of ATP at 30°C for 10 min. The UbcH7~Ub thioester intermediate was detected by Coomassie brilliant blue staining after separation using 15% non-reducing SDS-PAGE. (TIF) [file ppat.1005584.s002.tif]

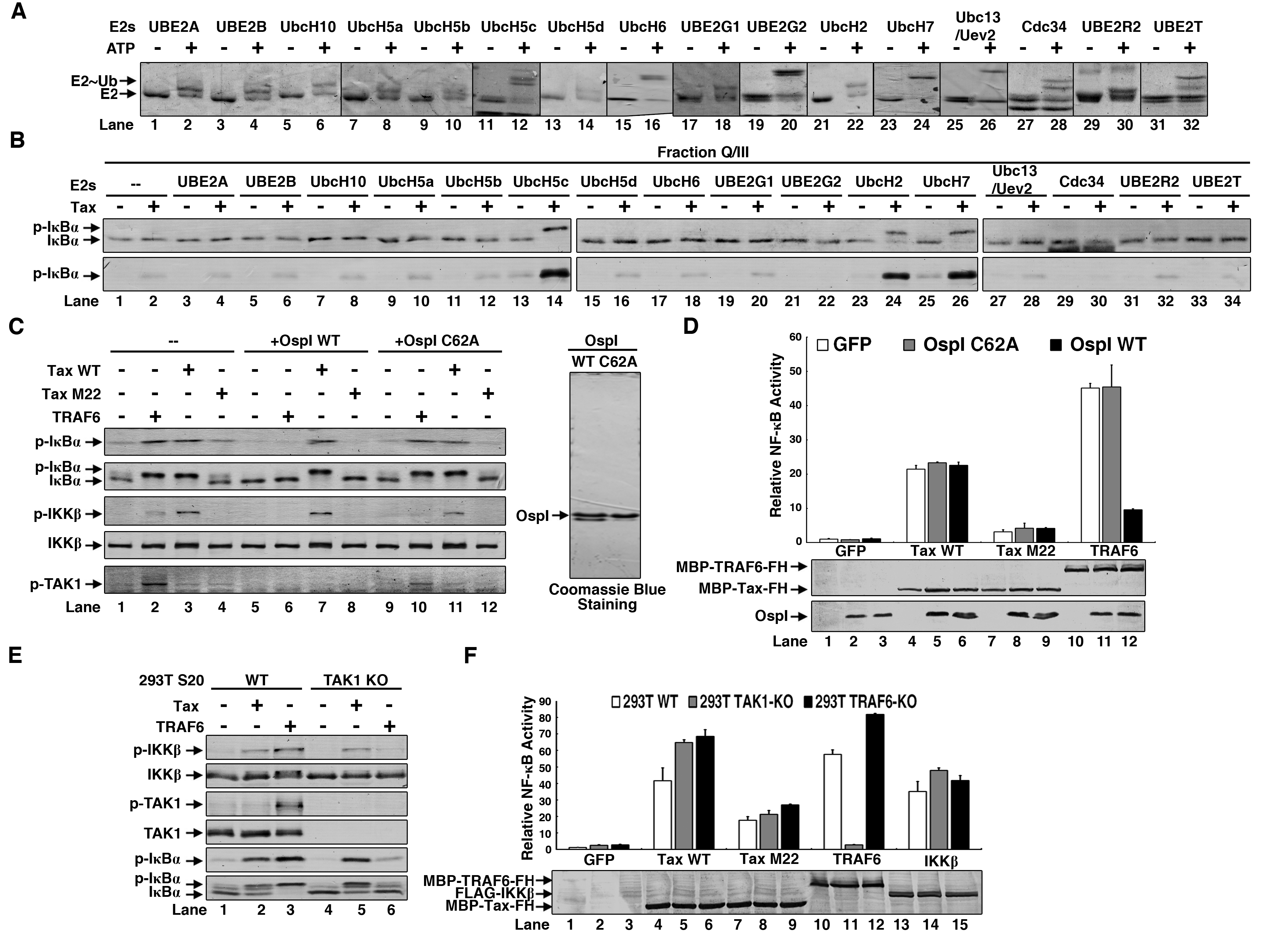

Supplement: S3 Fig — (A) Ubiquitin loading assay to verify E2 activity. The same ubiquitin loading assay as in S2D Fig was performed except UbcH7 was replaced with the indicated recombinant E2s. (B) Screening of E2s for Tax-dependent IKK activation in vitro. The same in vitro assay as in Fig 2D was performed except UbcH7 was replaced with the indicated recombinant E2s. (C) OspI doesn’t impair IKK activation by Tax in vitro. Jurkat T S100 was incubated with Tax WT, M22 or TRAF6, together with OspI WT or its enzymatic-dead mutant (C62A), in ATP buffer at 30°C for 1 h. Phosphorylation of TAK1, IKKβ and its substrate IκBα was detected by immunoblotting. (D) OspI doesn’t impair IKK activation by Tax in cells. 293T cells were transfected with OspI WT, C62A, or GFP as a control. After 6 hours, cells were transfected again with plasmids encoding Tax WT, M22 or TRAF6, together with a NF-κB firefly-luciferase reporter and a renilla-luciferase internal control. The luciferase activity was measured and normalized by renilla luciferase. (E) TAK1 is not involved in Tax-dependent IKK activation in vitro. Cell extracts (S20) of 293T or TAK1-deficient 293T cells were incubated with Tax WT or TRAF6, and ATP at 30°C for 1 h. Phosphorylation of TAK1, IKKβ and its substrate IκBα was detected by immunoblotting. (F) TAK1 and TRAF6 are not involved in Tax-dependent IKK activation in cells. 293T WT, TAK1 KO or TRAF6 KO cells were transfected with plasmids encoding Tax WT, M22, TRAF6, IKKβ or GFP, together with a NF-κB firefly-luciferase reporter. The luciferase activity was measured 24 h later and normalized by renilla luciferase. FH: FLAG-10xHis tag. (TIF) [file ppat.1005584.s003.tif]

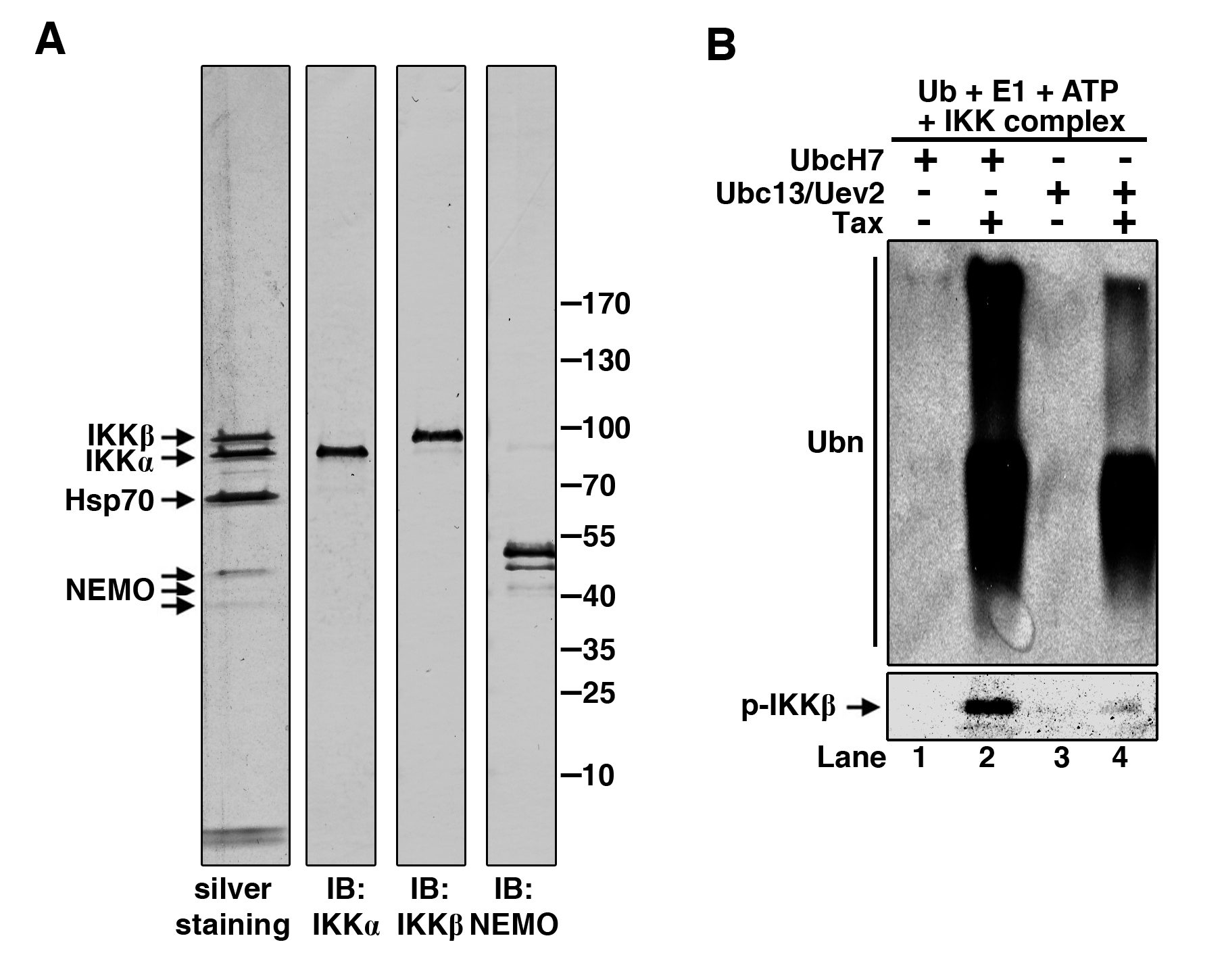

Supplement: S4 Fig — (A) Silver staining and immunoblotting of IKK complex purified as described in Materials and Methods. (B) Ubc13/Uev2 doesn’t support Tax-dependent IKK activation. The same assay as in Fig 4A was performed except UbcH7 was replaced by Ubc13/Uev2. (TIF) [file ppat.1005584.s004.tif]

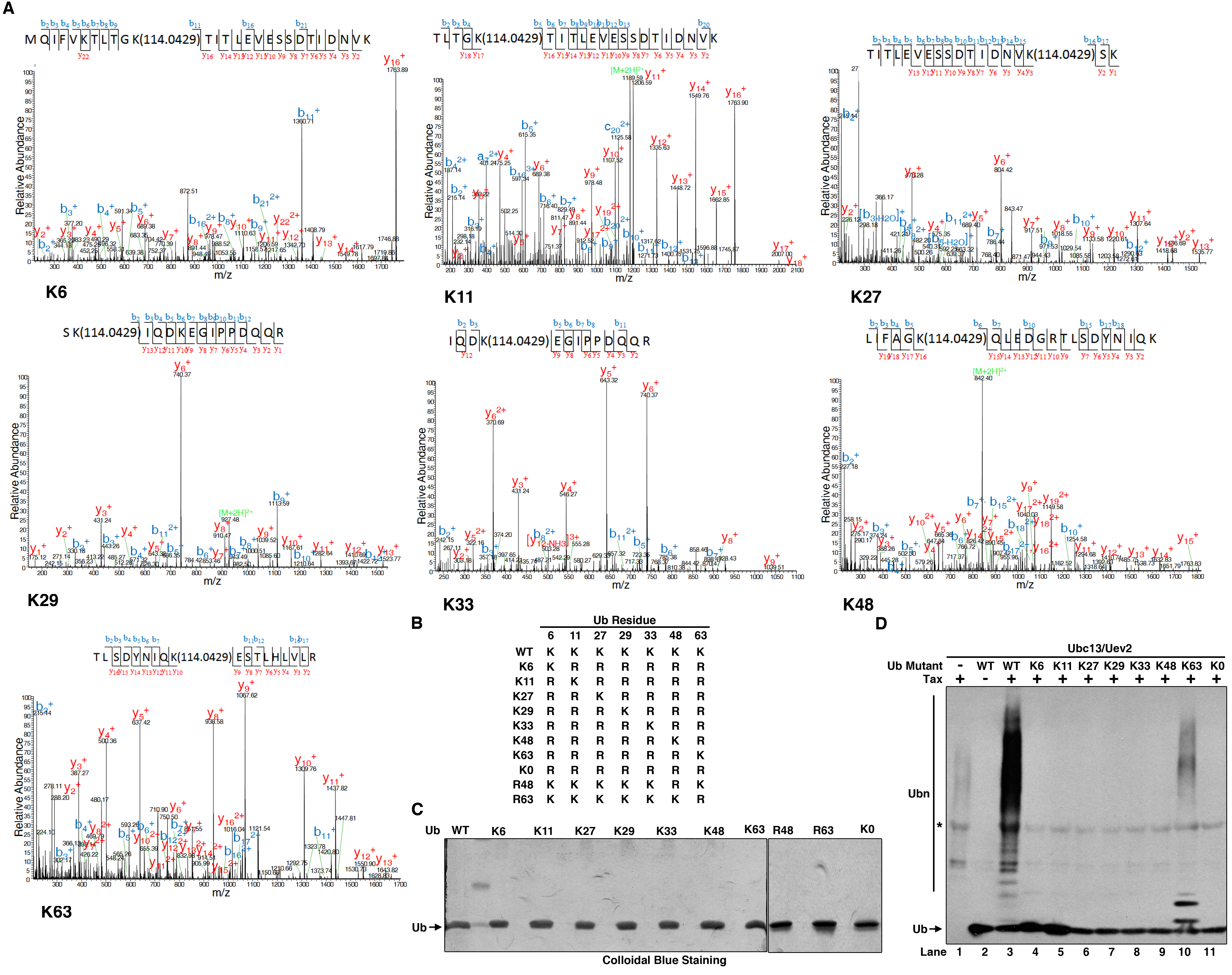

Supplement: S5 Fig — (A) Identification of Ub linkage assembled by Tax by using Thermo Q-exactive. A brief diagram was shown in Fig 6A. (B) Diagram of Ub mutants. (C) Colloidal blue staining of recombinant ubiquitin WT or mutant proteins. (D) Tax synthesizes K63-linkage polyUb chains with Ubc13/Uev2. The same assay as in Fig 6B was performed except Ubc13/Uev2 was used as the E2. (TIF) [file ppat.1005584.s005.tif]
